# Frequencies and TCR Repertoires of Human 2,4,6-Trinitrobenzenesulfonic Acid-specific T Cells

**DOI:** 10.3389/ftox.2022.827109

**Published:** 2022-02-22

**Authors:** Caterina Curato, Marina Aparicio-Soto, Franziska Riedel, Ingrun Wehl, Alev Basaran, Amro Abbas, Hermann-Josef Thierse, Andreas Luch, Katherina Siewert

**Affiliations:** ^1^ Dermatotoxicology Study Centre, Berlin, Germany; ^2^ Department of Chemical and Product Safety, German Federal Institute for Risk Assessment, Berlin, Germany; ^3^ German Rheumatism Research Center (DRFZ), Berlin, Germany; ^4^ Institute of Pharmacy, Freie Universität Berlin, Berlin, Germany

**Keywords:** activation-induced marker assay, allergic contact dermatitis, CDR3 amino acids, chemical sensitizer, high-throughput sequencing, t cell, T cell receptor, trinitrobenzene sulfonate

## Abstract

Allergic contact dermatitis is a widespread T cell-mediated inflammatory skin disease, but *in vitro* monitoring of chemical-specific T cells remains challenging. We here introduce short-term CD154/CD137 upregulation to monitor human T cell responses to the experimental sensitizer 2,4,6-trinitrobenzenesulfonic acid (TNBS). Peripheral blood mononuclear cells (PBMC) from healthy donor buffy coats were TNBS-modified and incubated with unmodified PBMC. After 5 and 16 h, we detected TNBS-specific activated CD154+CD4+ and CD137+CD8+ T cells by multi-parameter flow cytometry, respectively. Activated cells were sorted for restimulation and bulk T cell receptor (TCR) high-throughput sequencing (HTS). Stimulation with TNBS-modified cells (3 mM) induced CD154 expression on 0.04% of CD4+ and CD137 expression on 0.60% of CD8+ memory T cells, respectively (means, *n* = 11–17 donors). CD69 co-expression argued for TCR-mediated activation, which was further supported by TNBS-specific restimulation of 10/13 CD154+CD4+ and 11/15 CD137+CD8+ T cell clones and lines. Major histocompatibility complex (MHC) blocking antibodies prevented activation, illustrating MHC restriction. The high frequencies of TNBS-specific T cells were associated with distinct common changes in the TCR β-chain repertoire. We observed an overrepresentation of tryptophan and lysine in the complementarity determining regions 3 (CDR3) (*n* = 3–5 donors), indicating a preferential interaction of these amino acids with the TNBS-induced epitopes. In summary, the detection of TNBS-specific T cells by CD154/CD137 upregulation is a fast, comprehensive and quantitative method. Combined with TCR HTS, the mechanisms of chemical allergen recognition that underlie unusually frequent T cell activation can be assessed. In the future, this approach may be adapted to detect T cells activated by additional chemical sensitizers.

## Introduction

Chemical allergens bind to self-proteins to produce immunogenic epitopes recognized by T cell receptors (TCR). In the presence of appropriate stimulatory signals for the innate immune system (Martin and Esser, 2022), chemical-specific T cell activation leads to adaptive immune responses. Once sensitized, clinical symptoms, such as allergic contact dermatitis (ACD) occur, even after otherwise relatively harmless chemical exposures (Peiser et al., 2012; Esser and Martin, 2017; Martin et al., 2018). To date, only a few chemical-induced T cell epitopes, mainly for model proteins or peptides, have been elucidated (Martin et al., 2004; Thierse et al., 2005; Meng et al., 2018; Pichler, 2019; Riedel et al., 2021). Experimental hardships linked to inefficient epitope generation and the rarity of antigen-specific T cells have delayed the development of T cell-based assays (Martin et al., 2010; Ogese et al., 2020; Hammond et al., 2021; Riedel et al., 2021). However, alternative *in vitro* tests that include T cells are urgently needed for improved diagnosis and predictive purposes by worldwide regulatory authorities (Corsini et al., 2018).

One of the best-researched chemical model allergens is 2,4,6,-trinitrobenzenesulfonic acid (TNBS). TNBS and its lipophilic form, trinitrochlorobenzene (TNCB), promote T cell activation *in vitro* and *in vivo*, respectively (Gerberick et al., 1992; Moulon et al., 1993; Martin et al., 2000; Dietz et al., 2010; Richter et al., 2013). TNCB provides sufficient irritant signals by itself to sensitize germ-free mice (Martin et al., 2008) and has been used in murine colitis models (Antoniou et al., 2016). *In vitro*, TNBS binds covalently to free amino groups at a wide range of pH values by nucleophilic aromatic substitution (Habeeb, 1966; Freedman and Radda, 1968; Sarantonis et al., 1986). Thus, mainly antigenic trinitrophenyl (TNP) determinants are generated on accessible lysine residues of proteins or on their free N-terminal amino groups (Gevaert et al., 2003). The TNBS adductome, i.e., *in vivo* protein target sites for TNBS, remains unknown as for most chemical allergens (Ndreu et al., 2020).

Since the 1970ies, TNBS-modified cells, proteins or peptides have been employed to activate murine and human T cells. CD4+ and CD8+ T cells were shown to recognize TNP-modified peptides presented by proteins of the major histocompatibility complex (MHC) I and II, respectively, but not by TNP-modified MHC proteins themselves (von Bonin et al., 1992; Kohler et al., 1995). In H-2k^b^ C57BL/6 mice, CD8+ T cells mainly interact with TNP moieties at peptide position P4 independent from the amino acid sequence of the carrier peptide. A second set of TCR binds TNP-modified lysine at position P7 in combination with additional unmodified amino acids at positions P3 and P4 (Martin et al., 1993; Martin et al., 2003). TNBS-induced epitopes seem to activate an unusually large fraction of murine T cells (Hamann et al., 1983; Iglesias et al., 1992; Kohler et al., 1995; Martin et al., 2003). Recently, T cell priming assays confirmed the presence of TNBS-specific CD4+ and CD8+ naive T cells in humans, while exact frequencies remain elusive (Dietz et al., 2010; Richter et al., 2013).

High frequencies of murine TNBS-specific T cells and the analysis of TNBS-specific T cell clones led to speculations on a preferential interaction of TNBS-induced T cell epitopes with common TCR elements. The extensive diversity of TCR (≥100 × 10^6^TCR per individual) (Robins et al., 2009) is generated through V(D)J gene recombination and junctional random nucleotide insertions and deletions that yield to the complementarity determining region (CDR) 3. The CDR3 is mainly responsible for protein antigen binding (Rudolph et al., 2006). Possible interactions with TNBS include binding to certain TCR gene segments or to amino acids in the CDR3 (Hochgeschwender et al., 1986; Hochgeschwender et al., 1987; Kempkes et al., 1991; Martin et al., 1995). So far, bulk T cell analysis did not confirm a bias in TCR β-chain segment use for murine TNBS-specific CD8+ T cells (Martin et al., 2003). Technological advances over the last decades now enable the exploration of TCR repertoires by high-throughput sequencing (HTS) (Britanova et al., 2014; Aparicio-Soto et al., 2020; Barennes et al., 2021).

We here introduce a new short-term activation-induced marker (AIM) assay based on CD154 (CD40L) and CD137 (4-1BB) upregulation to quantify TNBS-specific CD4+ and CD8+ T cells. Both activation markers have been used for the detection of protein-specific T cells (Frentsch et al., 2005; Wolfl et al., 2007; Wehler et al., 2008; Bacher et al., 2013; Reiss et al., 2017; Elias et al., 2020; Saggau et al., 2021). TNBS-specific T cells were isolated for *in vitro* restimulation and TCR HTS. This proof-of-principle study elucidates frequencies and TCR repertoires of human TNBS-specific CD4+ and CD8+ T cells.

## Materials and Methods

### Blood Samples and Isolation of Peripheral Blood Mononuclear Cells

Since TNBS is an experimental allergen, patient samples are not available. We used buffy coats from healthy, most likely non-allergic, donors (German Red Cross, Supplementary Table S1). Buffy coats (∼80 ml) were diluted with an equal volume of MACS buffer (Miltenyi) and peripheral blood mononuclear cells (PBMC) were isolated by standard density gradient centrifugation with Ficoll Paque Plus (GE Healthcare), as described (Aparicio-Soto et al., 2020). Cells were cultured in complete RPMI 1640-based T cell media (TCM, see Supplementary Methods). Cell numbers were determined with a CASY Cell Counter (OMNI Life Science). Experiments were conducted according to the current version of the declaration of Helsinki (Charité's ethics committee, vote EA1_217_19).

### Antigen Presenting Cell Preparation

PBMC were labeled with carboxyfluorescein succinimidyl ester (CFSE) (0.5 µM in PBS, CellTrace™ CFSE, Thermofisher) for 15 min. After CFSE labeling, PBMC were incubated with phosphate buffered saline (PBS, control) or TNBS (0.05–25 mM in PBS) for 10 min at 37°C and washed extensively, as described to yield modified PBMC (Shearer et al., 1974; Richter et al., 2013).

### T Cell Antigen Stimulation Assay

Modified PBMC and unmodified “responder” PBMC were cultured in a 1:1 ratio at a density of 2.5 × 10^6^ cells/cm^2^ in flat bottom tissue culture plates (TPP) at 37°C in a 5% CO^2^ >95% humidified atmosphere. To prevent ligand-induced downregulation of CD154, CD40 blocking antibody was added (Yellin et al., 1994; Frentsch et al., 2005). In some experiments, staphylococcal enterotoxin B (SEB) (1 μg/ml) or phorbol myristate acetate-Ionomycin (PMA-I) (10 ng/ml and 1 μg/ml, all Sigma-Aldrich) were used as positive controls for T cell activation. For MHC blocking experiments, antibodies were added 30 min prior to antigen stimulation. T cell assays were performed for 5 and/or 16 h.

### Antibody Staining and Flow Cytometry Analysis

Cells were stained for 20 min at room temperature with different combinations of fluorochrome-conjugated antibodies and dead cell stain (see Supplementary Methods). Analysis and sorting were performed on a BD FACSAria III with BD Diva7.0 Software (BD). Data were further analyzed in FlowJo (V.10.7.1, BD Biosciences).

### Cell Sorting, *in vitro* Expansion and T Cell Restimulation

TNBS-specific CD154+CD4+ and CD137+CD8+ memory T cells (single cells and lines) were sorted in single cell mode into 200 µL TCM supplemented with 20% human AB serum, 300 U/ml interleukin (IL)-2 (IS grade), 30 ng/ml anti-CD3 (both Miltenyi Biotec) and ∼200.000 allogeneic mitomycin C-treated PBMC as feeder cells on 96-well flat-bottom plates. After one week, half of the media was replaced with fresh TCM supplemented with 15% human AB serum and 200 U/ml IL-2. Cells were further expanded on 48-well plates pre-coated with anti-CD3 antibody (OKT-3) (1 μg/ml in PBS for 1 h at 37°C) and, if required for CD4 clones, 10.000 CD3/CD28 coated beads per well (Dynabeads Human T-Activator, Thermo Fisher Scientific). Before restimulation, clones rested for ∼3 days in TCM supplemented with 10% human AB serum (without IL-2 and/or CD3/CD28 beads). Restimulation was performed with cryopreserved, thawed and overnight rested autologous CD3-depleted PBMC (human CD3 MicroBeads and LD columns, Miltenyi Biotech) modified with TNBS (3 mM) or PBS (control) in 384-well plates. Conditions were the same as used for bulk culture stimulation assays, except for the additional administration of co-stimulatory CD28 antibody (1 μg/ml).

### T Cell Receptor High-Throughput Sequencing and Data Analysis

TNBS-specific CD154+CD4+ memory T cells and CD137+CD8+ memory T cells were sorted in purity mode directly into 1 ml buffer RLT (Qiagen) and stored at −80°C. RNA was extracted with RNeasy Micro Kit (Qiagen). Reverse transcription with the introduction of a unique molecular identifier (UMI)-containing SMART adapter, PCR amplification, library preparation and Illumina sequencing for TCR αβ chains were performed as described (Aparicio-Soto et al., 2020). PCR products were purified with QIAquick PCR Purification Kit (Qiagen), pooled, size selected to ∼300–600 bp with beads (0.65 ratio, CleanPCRNA kit, GC Biotech), and sequencing adapters were annealed using TruSeq DNA PCR-Free Low Throughput Library Prep Kit (Illumina). Libraries were quantified using a Qubit device (Qubit™ dsDNA HS Assay Kit, Thermo Fisher Scientific). Sequencing was done with MiSeq Reagent Kit v3 (2 × 250 bp paired-end sequencing, Illumina). Raw data were demultiplexed and error corrected with MIGEC (v1.2.9; over sequencing threshold ≥4 reads per UMI) (Shugay et al., 2014). TCR were extracted with MIXCR (v3.0.13; library repseqio. v1.6) (Bolotin et al., 2015) and sequences with identical V-, (D-), and J-gene segments as well as CDR3 nucleotide sequence were considered as one TCR clonotype. Further analysis was performed with VDJtools v1.2.1 (e.g., removal of non-functional clonotypes) (Shugay et al., 2015) and own custom Python software (available upon reasonable request). GraphPad Prism 8 was used for visualization. International ImMunoGeneTics information system nomenclature (IMGT) is used throughout the manuscript.

### Quantification and Statistical Analysis

Statistical tests and n values are specified in the respective figure legends. A *p* value < 0.05 was considered statistically significant. For flow cytometry data, statistical analysis was only performed with populations containing at least 20 cells.

### Supplementary Methods and Data Availability

Further experimental details can be found in the Supplementary Material, Supplementary Methods. TCR sequencing data are available at European Nucleotide Archive (ENA) under study number PRJEB49381 (https://www.ebi.ac.uk/ena/data/view/PRJEB49381).

## Results

### Trinitrobenzene Sulfonate Efficiently Modifies Peripheral Blood Mononuclear Cells

**FIGURE 1:**
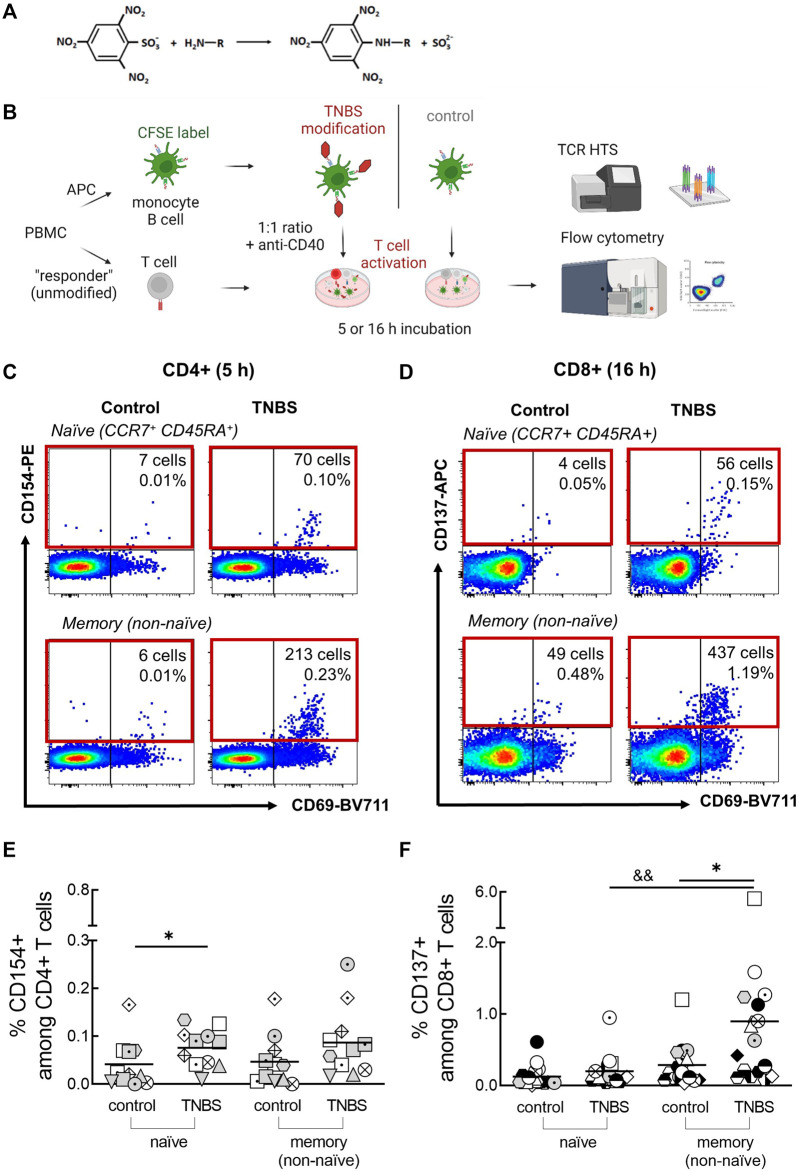
Detection of TNBS-specific T cells by CD154/CD137 upregulation. **(A)** TNBS reaction scheme. TNBS mainly binds to primary amino groups. **(B)** Assay setup. PBMC were labeled with CFSE, modified with PBS or TNBS and mixed as APC with unmodified autologous “responder” PBMC. CD4+ and CD8+ T cell activation was detected by flow cytometry after 5 and 16 h, respectively, and activated cells were sorted for TCR HTS. Created with BioRender.com. **(C,D)** Representative dot plots showing numbers and frequencies of CD154+CD4+ [**(C)**, MLB20] and CD137+CD8+ [**(D)**, MLB31] naive and memory T cells after incubation with PBS- (control) or TNBS-modified APC. **(E,F)** Summarized frequencies of TNBS-specific CD4+ **(E)**, *n* = 11) and CD8+ T cells [**(F)**, *n* = 17]. Each symbol represents one donor (buffy coats, Supplementary Table S1). The gating strategy can be found in Supplementary Figure S4. Horizontal lines indicate the mean values. Statistical significances were determined by non-parametric Mann-Whitney *t*-test (*p < 0.05 vs. control; ^&&^ *p* < 0.01 vs. naïve TNBS).

TNBS produces antigenic TNP-determinants mainly by binding to free ε-amino groups of lysine in peptides presented by MHC proteins on the surface of antigen presenting cells (APC, Figure 1A) (Weltzien et al., 1996). As APC, we used autologous PBMC. We monitored extracellular TNBS surface modification by anti-TNP staining. TNBS modification was reproducible for different TNBS lots (Supplementary Figure S1A). Compared to T cells, monocytes and B cells showed an increased shift in the mean fluorescence intensity. This indicates the modification of a larger number of cell surface proteins, which may reflect the generation of more T cell epitopes (Supplementary Figure S1B). TNBS concentrations ranging between 0.8 and 3.1 mM resulted in the brightest anti-TNP stain while input cell numbers were preserved.

### Trinitrobenzene Sulfonate-Modified Antigen Presenting Cells are Non-toxic in CD154/CD137 Upregulation Assays

To exclude toxic effects of TNBS on PBMC in CD154/CD137 upregulation assays, we monitored total live cell numbers and T cell activation capacity to the superantigen Staphylococcal enterotoxin B (SEB). SEB activates a large fraction of T cells independent of their antigen-specificity and thus it superimposes any TNBS-specific T cell activation. We tested a two-fold dilution series of TNBS concentrations (25–0.05 mM) for APC modification and analyzed both TNBS-labeled PBMC (APC) and unlabeled “responder” PBMC (Figure 1B).

Among TNBS-modified PBMC, toxic effects occurred after modification with 12.5 mM or higher TNBS concentrations. This was exemplified by a decline in total CD4+ and CD8+ memory T cell numbers and reduced CD154/CD137 upregulation capacity upon SEB stimulation after 5 or 16 h of antigen stimulation (Supplementary Figures S2, S3). Similar data were obtained for naive T cell, CD14+ monocytes and CD19+ B cells (data not shown). T cells from TNBS-modified PBMC can respond to SEB and remain functional upon modification with up to 6.3 mM TNBS. However, we did not further analyze activated T cells within the APC fraction.

T cells from unmodified “responder” PBMC did not show sign of toxicity upon incubation with TNBS-modified cells (Supplementary Figures S2, S3). SEB-induced T cell activation stayed constant even if APC numbers were drastically reduced after modification with 12.5 mM TNBS or 25 mM TNBS at the end of the incubation period. Thus, harsh conditions may ensure efficient *in vitro* T cell epitope formation in this experimental setup.

We chose 3 mM TNBS as standard concentration for APC preparation in CD154/CD137 upregulation assays. This concentration was the highest with bright anti-TNP stain and in the non-toxic range for TNBS-modified APC. In addition, most studies in the literature use 3 mM TNBS (Dietz et al., 2010; Richter et al., 2013).

### Trinitrobenzene Sulfonate-specific Human T Cells are Frequent

The main aim of this study was to determine frequencies and TCR repertoires of human TNBS-specific CD4+ and CD8+ T cells using CD154 and CD137 upregulation, respectively. The experimental setup is illustrated in Figure 1B. PBMC were CFSE-labeled, TNBS or control (PBS)-modified and mixed as APC in a 1:1 ratio with non-modified “responder” PBMC in the presence of anti-CD40 antibody to prevent ligand-induced down-regulation of CD154 (Yellin et al., 1994; Frentsch et al., 2005). After 5 or 16 h of antigen stimulation, cells were stained and frequencies of CD154+CD4+ and CD137+CD8+ T cells were determined by multi-parameter flow cytometry. The incubation times were chosen according to the required times for maximum induced CD154 (∼5 h) and CD137 expression (∼16–24 h) (Frentsch et al., 2005; Wolfl et al., 2007; Wehler et al., 2008; Aparicio-Soto et al., 2020). The gating strategy to detect expression of both activation markers is depicted in Supplementary Figure S4.

Background expression of CD154 was negligible on control-stimulated CD4+ T cells (0.04% ± 0.05 and 0.05% ± 0.05%, naive and memory cells, respectively; means ± standard deviation, Supplementary Table S1). Incubation with TNBS-modified APC substantially increased frequencies to 0.08% ± 0.04% (naive) and 0.09% ± 0.07% (memory) CD154+CD4+ T cells (*n* = 11 buffy coats, Figures 1C,E). Signals were similar after 16 h of antigen stimulation (0.08% ± 0.1 and 0.13% ± 0.18%, Supplementary Figure S5A). A constant CD154 signal over this timeframe was expected from earlier studies (Frentsch et al., 2005; Aparicio-Soto et al., 2020). However, some buffy coats showed an increased signal in 16 h experiments compared to 5 h experiments, e.g., LMB1, MLB31. Increased 16 h signals were accompanied by high frequencies of TNBS-specific CD137+CD8+ T cells and may thus be cytokine-induced (Supplementary Figure S5A) (Skov et al., 2000). To avoid the analysis of activated bystander CD4+ T cells, we analyzed TCR repertoires and T cell clones from 5 h experiments. In summary, we observed 0.04% TNBS-specific CD154+CD4+ naive and memory T cells in human buffy coats.

Background expression of CD137 on CD8+ T cells was higher and more variable among different buffy coats compared to CD154 (Figures 1D,F; Supplementary Table S1). After 16 h of stimulation with PBS-modified APC (control), we observed 0.13% ± 0.15 and 0.29% ± 0.28% CD137+CD8+ naive and memory T cells, respectively. Stimulation with TNBS-modified APC resulted in 0.20% ± 0.21 and 0.90% ± 1.2% of CD137+CD8+ T cells (*n* = 17 buffy coats, Figure 1E). A small signal was already visible after 5 h, as expected from the slower expression kinetics of CD137 compared to CD154 (0.1% ± 0.1 and 0.4% ± 0.2%, *n* = 8 buffy coats, Supplementary Figure S5B). TNBS-induced CD137+CD8+ T cells were rarer among the naive compared to the memory compartment. In summary, we detected 0.6% TNBS-specific CD137+CD8+ memory T cells in human buffy coats.

CD69 is another activation marker that is upregulated by TCR engagement (Testi et al., 1989; Yamashita et al., 1993; Beeler et al., 2008). However, background expression of CD69 on control-stimulated T cells was very high (Supplementary Figures S5C, D). We only detected signals for TNBS-specific CD69+CD8+ memory T cells (Supplementary Figures S5C, D). In summary, CD69 was less suited as a specific activation marker compared to CD154 (for CD4+ T cells) and CD137 (for CD8+ T cells).

Early CD137 expression on CD4+ T cells can be used to identify antigen-specific regulatory T cells (Schoenbrunn et al., 2012; Saggau et al., 2021). However, we observed a rather high background expression of CD137 on CD4+ T cells and did not detect TNBS-specific regulatory T cells (Supplementary Figure S5E). Similarly, we did not detect TNBS-specific CD154+CD8+ T cells in most buffy coats (data not shown), since only a minority of CD8+ T cells expresses this activation marker (Frentsch et al., 2013).

### T Cell Receptor-Mediated CD154/CD137 Upregulation by Trinitrobenzene Sulfonate-specific T Cells

We confirmed TCR-mediated CD154/CD137 upregulation on TNBS-specific T cells by analyzing CD69 co-expression, restimulation of activated T cells and effects of MHC blocking antibodies.

High levels of CD69 co-expression were observed among TNBS-specific CD154+CD4+ and CD137+CD8+ naive and memory T cells (Supplementary Figure S6A, B; Supplementary Table S1). CD69 co-expression is a well-established indicator for TCR-mediated activation for protein and nickel-specific CD4+ T cells (Bacher et al., 2013; Aparicio-Soto et al., 2020). Among TNBS-specific CD8+ T cells, CD69 co-expression was less frequent (74 and 64%, means of naive and memory T cells, respectively, *n* = 11 buffy coats) compared to the co-expression in TNBS-specific CD4+ T cells (71 and 82%, *n* = 6). CD69 co-expression among control activated T cells likely indicates *ex vivo* activation or auto-reactivity and was not further considered. In summary, co-expression of the CD69 activation marker among the majority of CD154+ or CD137+ T cells indicates antigen-specificity and TCR-mediated TNBS recognition.

**FIGURE 2:**
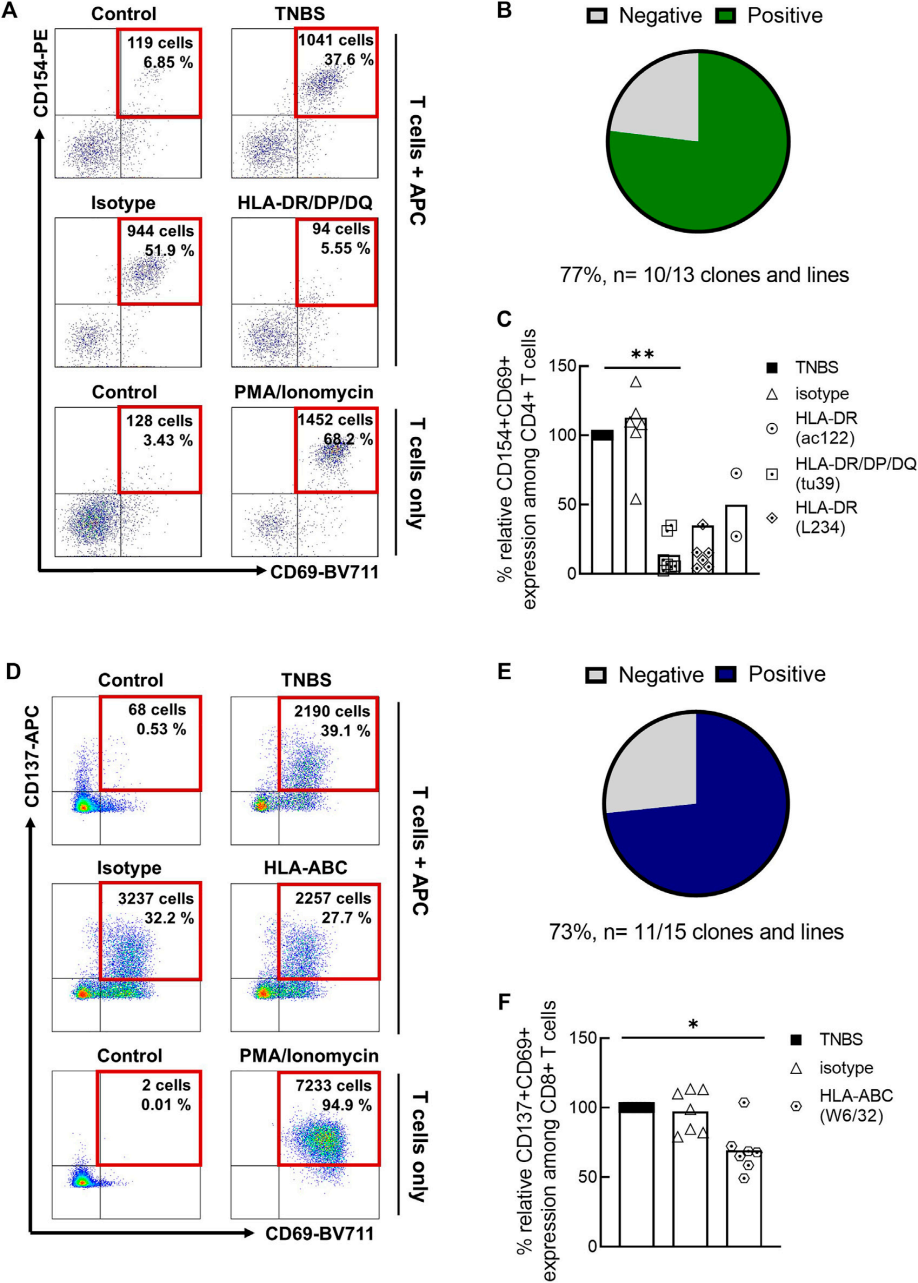
Restimulation of TNBS-specific T cells. TNBS-specific CD154+CD4+ or CD137+CD8+ memory T cells were sorted from 5 to 16 h experiments, respectively. After *in vitro* expansion, cells were restimulated with TNBS-modified CD3-depleted autologous PBMC. **(A,D)** Representative dot plots for one CD4+ T cell clone [CACB3, **(A)**] and one CD8+ T cell clone [MLB42a, **(D)**]. As a negative control, T cells were incubated with PBS-modified APC (control). PMA/iono stimulation was used as a positive control for T cell reactivity. Gated on live, CD3+, single, CD4+ or CD8+ T cells. **(B,E)** Summary of restimulation experiments for TNBS-specific CD4+ **(B)** and CD8+ **(E)** T cell clones and lines (listed in Supplementary Table S2). **(C,F)** MHC blocking experiments. MHC II or MHC I blocking antibodies were added to APC 30 min before incubation with T cells. Shown is the relative inhibition of induced CD154 and CD137 expression on TNBS-specific CD4+ and CD8+ T cell clones and lines, respectively. Signals for stimulation with TNBS-modified APC without antibodies were set to 100%. White bars represent the mean values. Statistical significances were determined by one-way non-parametric ANOVA analysis (Kruskal-Wallis) with Dunn’s test as post-hoc test (*p < 0.05; **p < 0.01 vs. TNBS).

An independent method to investigate antigen-specificity is the sorting of TNBS-activated T cells, *in vitro* expansion and restimulation (Figure 2; Supplementary Table S2). As APC, we used autologous TNBS or control-modified CD3-depleted PBMC. Among TNBS-specific CD4+ T cell clones and lines, 10/13 (77%) responded to TNBS restimulation with CD154 upregulation (Figures 2A,B). The presence of different MHC II blocking antibody clones prevented T cell activation, further confirming antigen-specificity and conventional MHC II restriction for TNBS-specific CD4+ T cells (Figure 2C). Results were similar for TNBS-specific CD137+CD8+ memory T cell clones and lines. 11 out of 15 (73%) TNBS-specific clones or lines were activated by TNBS-modified APC (Figures 2D,E) and activation was reduced in the presence of an MHC I blocking but not isotype control antibody clones (Figure 2F).

Taken together, CD69 co-expression, restimulation of TNBS-specific T cell clones and lines and MHC block experiments confirm that a large fraction of TNBS-activated CD154+CD4+ and CD137+CD8+ T cells recognize TNBS-induced epitopes *via* their TCR.

### Trinitrobenzene Sulfonate-specific CD4+ and CD8+ T Cell Receptor Repertoires Show Distinct Features

Although TNBS has been used extensively to study T cell activation, it remained unclear whether TNBS-induced epitopes preferentially interact with TCR gene segments or with specific amino acids in the CDR3. To address this research objective, we sorted ∼450–2500 TNBS-specific CD154+CD4+ and CD137+CD8+ memory T cells from several buffy coats and analyzed their TCR by HTS. For comparison, we also analyzed randomly sorted CD4+ and CD8+ memory T cells. Using an RNA and UMI-based protocol, we obtained transcribed TCR α- and β-chain cDNA (counts, Supplementary Table S3) for each sample, distributed among different TCR clonotypes (diversity, Supplementary Table S3). Results on TCR repertoire analysis are shown for TCR clonotypes (diversity percentages) because there is no exposure to TNBS and clonotype expansions are not linked to a TNBS allergy. For some samples, relatively small repertories were analyzed and coincidental clonotype (count) frequencies of expanded TNBS-specific cross-reactive memory T cells could influence the results.

Gene segment use by random TCR α- and β-chains varied considerably among buffy coats (Supplementary Figure S7). We observed a relatively small common increase of the TRBV20-1 gene segment among TNBS-specific CD4+ T cells (8% of random compared to 13% of TNBS-specific TCR, adj. *p*-value 0.004; Supplementary Figures S7B, D). Among TNBS-specific CD8+ T cells, changes of similar magnitude were observed for several V-gene segments, e.g., TRAV26-1 and TRBV28 (adj. *p*-value n.s.; Supplementary Figures S7G, H). Changes in J-gene segment use were similar but not further considered. Antigen recognition is mainly mediated by the three CDR regions with the CDR1 and CDR2 occurring in the V-gene segments. In general, we found no hints that TNBS-induced T cell activation is based on the interaction with one major TCR gene segment.

**FIGURE 3:**
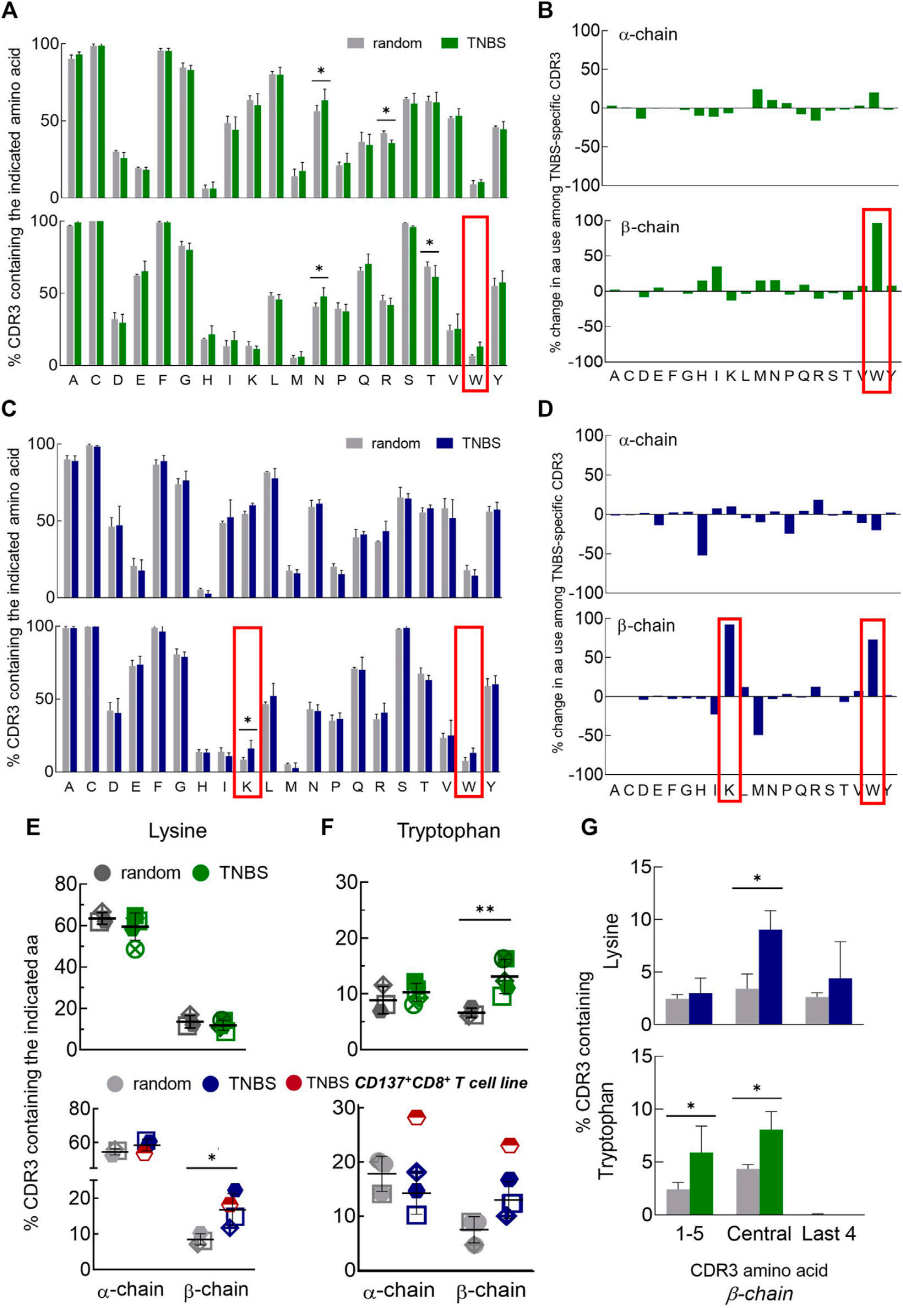
Changes in CDR3 amino acid composition among TNBS-specific TCR. TNBS-specific CD154+CD4+ and CD137+CD8+ memory T cells were sorted from 5 to 16 h experiments, respectively, and their TCR were sequenced (Supplementary Table S3). Random CD4+ and CD8+ memory T cells from the same buffy coats served as control. **(A,C)** CDR3 amino acid compositions. The bar charts depict the occurrence of individual amino acids in the CDR3 of TCR α- (upper panel) and β-chains (lower panel) in single letter code for random (white) and TNBS-specific CD154+CD4+ [green, **(A)**] or CD137+CD8+ [blue, **(C)**] T cells, respectively (mean values, TCR diversity, *n* = 3–5). The first C and last F of the CDR3 have been included in the analysis. The red box indicates the most prominent changes. **(B,D)** Percent change in amino acid use. Plotted are relative changes in CDR3 amino acid use among TCR α- and β-chains of TNBS-specific compared to random TCR. Data are based on the mean values shown in **(A,C)**. **(E,F)** Individual donor values showing the overrepresentation of lysine **(E)** and tryptophan **(F)** among TNBS-specific TCR. The red symbol represents data from a TNBS-specific CD137+CD8+ memory T cell line (MLB42a_CD8_1C-50, not included in the statistics). **(G)** Positional CDR3 amino acid analysis. The graphs depict lysine (upper panel) and tryptophan (lower panel) occurrence according to their locations in the β-chain of TNBS-specific TCR (see also Supplementary Figure S8). Statistical significance was determined by multiple *t*-test **(A,C,G)** or two-way ANOVA **(E,F)**. Multiple comparisons were corrected according to the Holm-Sidak’s method **(E-G)** (*n* = 3–5, *p < 0.05, **p < 0.01 vs. random).

Most interactions for conventional antigen recognition by TCR occur in the CDR3 region. To assess the possible involvement of certain amino acids in TNBS-induced epitope recognition, we analyzed the amino acid composition of TNBS-specific and random TCR (Figure 3). Inter-individual variations in the CDR3 amino acid composition were lower than in the gene segment use analysis. The amino acid composition differs for TCR α- and β-chains, but these differences were similar for CD4+ and CD8+ T cells (Figures 3A–D). For instance, isoleucine (I) and lysine (K) residues are more common among TCR α-chains (CD4: I 44% vs. 18% and K 60% vs. 11%, CD8: I 53% vs. 11%, K 60% vs. 16%, α- vs. β-chain, respectively) while histidine (H) occurs more frequently among TCR β-chains (CD4: 6 vs. 22%, CD8: 3% *vs.* 13%, α- vs. β-chain, respectively) (Figures 3A,C).

Comparing TNBS-specific and random TCR, nearly all CDR3 amino acids were similarly represented. Exceptions were an increased frequency of TCR β-chains with lysines (K) in TNBS-specific CD8+ T cells and tryptophans (W) in TNBS-specific CD4+ and CD8+ T cells (Figures 3B,D–F). Lysine overrepresentation was focused on central amino acids in the β-chain CDR3 and also occurred in the central amino acids among TNBS-specific CD4+ T cells (Figure 3G, Supplementary Figure S8A). The most pronounced changes in tryptophan use occurred among the central and N-terminal CDR3 amino acids (Figure 3G, Supplementary Figure S8B). We further observed a statistically significant increase in the use of asparagine (N), while arginine (R) and threonine (T) use decreased among the TCR chains of CD4+ T cells (Figure 3A). For asparagine (N), we could not observe any difference within the individual CDR3 amino acid positions probably due to the prominent expression by random TCR (data not shown). We performed further analysis of the exact amino acid position on the CDR3 of TCR α- and β-chains (Supplementary Figures S9). This analysis illustrates the occurrence of conserved flanking amino acids at the beginning of the CDR3 (in 5′-3′ direction, Supplementary Figures S9A, B). We found that lysine in position 7 of the TCR β-chain CDR3 may be favorable for TNBS-induced epitope recognition (Supplementary Figure S9C). Tryptophan locates closer to the N-terminus of the TCR β-chains. Position 3 for CD4+ T cells and position 5 for CD8+T cells may be favorable for TNBS-induced epitope recognition (Supplementary Figure S9D). TCR α-chains were in general more reluctant at changes in the composition and location of CDR3 amino acids (Figure 3, Supplementary Figures S8, S9).

In summary, these data confirmed that although no major TCR gene segments are involved in the recognition of TNBS, the high frequencies of TNBS-specific T cells are associated with distinct common changes in the CDR3 amino acid composition mainly in the TCR β-chain repertoire.

## Discussion

The *in vitro* monitoring of chemical-specific T cells remains challenging. We here introduce short-term CD154/CD137 upregulation for the detection of human TNBS-specific CD4+ and CD8+ naive and memory T cells. We combine a well-established technique for the generation of TNBS-induced T cell epitopes with recently developed AIM assays for the detection of protein antigen and nickel-specific T cells (Shearer et al., 1974; Frentsch et al., 2005; Wolfl et al., 2007; Wehler et al., 2008; Bacher et al., 2013; Richter et al., 2013; Aparicio-Soto et al., 2020).

Due to its reactivity, TNBS covalently binds to free amino groups within 10 min, including ε-amino groups of lysine-containing peptides presented by MHC proteins on the cell surface. Using an anti-TNP antibody staining, we showed an efficient and reproducible cell surface modification, especially for monocytes and B cells, which likely serve as APC in PBMC-based T cell assays. Unfortunately, the availability of hapten-specific antibodies is rather an exception than a rule, making it impossible to monitor cell surface modifications for most chemical allergens.

Incubation of responder PBMC with TNBS-modified autologous PBMC induced an average of ∼0.04% TNBS-specific CD154+CD4+ T cells. We observed some individual donor variations, illustrating the need to analyze several donors in T cell based assays. A human leukocyte antigen (HLA) allele association is unknown for TNBS-induced T cell activation. TCR-mediated activation and thus antigen-specificity was confirmed by analyzing the co-expression of CD69 and by restimulation of T cell clones and lines. MHC blocking antibodies prevented activation, proving MHC restriction. We observed equal frequencies of TNBS-specific CD154+CD4+ T cells in the naive and memory compartment, comparable to our study on nickel-specific CD4+ naive and memory T cells from non-allergic individuals (Aparicio-Soto et al., 2020). Thus, cross-reactivity or poly-specificity to TNBS- or nickel-induced T cell epitopes is an intrinsic feature of human TCR repertoires (Bechara et al., 2021; Riedel et al., 2021).

Noteworthy, CD154 function has been associated with systemic autoimmunity and likely contributes to immune cell reactivity in human contact allergy (Mehling et al., 2001; Schonbeck and Libby, 2001; Beissert et al., 2006; Caproni et al., 2007). The interplay between keratinocytes and T cells may affect transcriptional induction of selected genes such as PD1 and CD40 ligands, TNF-α and caspase-1, as shown in co-cultures with CD8+ T cells (Rauschenberger et al., 2019).

TNBS-specific CD137+CD8+ memory T cells were ∼10-times more frequent compared to TNBS-specific CD154+CD4+ T cells. The lower frequency of naive TNBS-specific CD8+ T cells is most likely due to a less efficient CD137 upregulation by naive CD8+ T cells (Wolfl et al., 2007). As for CD154+CD4+ T cells, most TNBS-activated CD137+CD8+ memory T cells seem activated *via* their TCR given prominent CD69 co-expression, efficient restimulation of clones and lines from different buffy coats, reduced activation in the presence of MHC blocking antibodies and the occurrence of common TCR repertoire features. Similar to the results of this human study, high frequencies of TNBS-specific CD8+ T cells have been found in mice (Hamann et al., 1983; Iglesias et al., 1992; Martin et al., 2003). Usually, frequencies of protein antigen-specific T cells are orders of magnitude lower in the absence of exposure and adaptive immune responses, e.g., ranging from 1–100 cells per 10 million T cells in the naive or cross-reactive memory compartments (Bacher et al., 2013; Su et al., 2013). Thus, TNBS-induced T cell epitopes interact with unusually large fractions of T cells, similar to nickel ions (Aparicio-Soto et al., 2020), which prompted investigations of the involved TCR.

We here report a first comprehensive and unbiased TCR assessment of human TNBS-specific CD4+ and CD8+ memory T cells. Previous studies on murine TNBS-specific T cells suggested a strong association with certain TCR gene segments or CDR3 amino acids (Hochgeschwender et al., 1986; Hochgeschwender et al., 1987; Kempkes et al., 1991; Martin et al., 1995). However, we did not identify a single dominant feature among human TNBS-specific T cells but rather several moderate changes in the TCR repertoires indicating the existence of diverse mechanisms for TNBS-related epitope recognition. We observed a moderate, common and significant overrepresentation of TCR with the gene segment TRBV20 among TNBS-specific CD4+ T cells. However, effects were much less prominent compared to the overrepresentation of the TRAV9-2 gene segment among nickel-specific CD4+ T cells (Aparicio-Soto et al., 2020).

TNBS-specific TCR from CD4+ and CD8+ T cells more often contained a lysine and tryptophan in their β-chain CDR3 compared to random TCR, respectively. The involvement of tryptophan in the recognition of TNP-modified amino acids has also been described for antibodies (Little and Eisen, 1967). We did not observe any change in tryptophan or lysine use among the TCR of nickel or cytomegalovirus-specific CD4+ T cells in our prior studies, excluding general effects on the occurrence of these amino acids among antigen-specific TCR (Aparicio-Soto et al., 2020). Instead, TCR of nickel-specific T cells showed a strong overrepresentation of CDR3 histidine (Aparicio-Soto et al., 2020). Interestingly, TNBS-associated TCR repertoire changes mainly occurred in the TCR β-chain while the overrepresentation of histidine among the TCR of nickel-specific CD4+ T cells occurred among both α- and β-chains (Aparicio-Soto et al., 2020). The differences in the general amino acid composition of α- and β-chain CDR3 illustrate the importance of a fine-grained TCR analysis from T cell subpopulations and the sequencing of appropriate control repertoires in order to identify the mechanisms of TCR-mediated chemical allergen recognition.

The TNP moiety may be a dominant interaction partner in T cell activation, as shown for murine TNBS-specific TCR (Burakoff et al., 1976; Martin et al., 1995; Martin et al., 2003). Especially for carrier-peptide independent chemical allergen recognition, PBMC-based assays may represent skin-derived T cell epitopes very well. For other TCR a lack of skin-derived T cell epitopes may prevent antigen-specific T cell activation in PBMC-based assays, as shown for some nickel-specific T cell clones (Kapsenberg et al., 1987). However, representative chemical-reactive T cell clonotypes can be identified by a PBMC-based T cell assay. TNBS-specific TCR were shown to be cross-reactive with dinitrobenzenesulfonic acid (DNBS)-induced T cell epitopes, once confounding effects of a lower or different DNBS reactivity had been excluded (Martin and Weltzien, 1994; Dietz et al., 2010). To fully elucidate details of the multimolecular interactions between chemical, TCR and peptide-MHC complexes, mutation or crystallization studies are needed. Of note, T cells can be activated by a single ligand (Sykulev et al., 1995; Huang et al., 2013), which renders the identification of T cell epitopes challenging until today.

So far, mainly interactions of chemicals with peptides presented by MHC proteins have been studied (Aparicio-Soto et al., 2021). Interactions with TCR residues remain unexplored although they have been theoretically addressed as “p-i TCR” concept (Pichler, 2019). For the drug and chemical allergen sulfamethoxazole, binding to TCR CDR2 and CDR3 regions has been modeled while a functional involvement in drug hypersensitivity remains unknown (Watkins and Pichler, 2013; Pichler, 2019). We here provide experimental evidence for this postulated direct interaction of chemical haptens with the TCR, similar to our recent study on nickel-specific CD4+ T cells.

In general, the “irritant” capacities of a chemical allergen, i.e., its activation of the innate immune system, has been linked to its sensitizing potency (Galbiati et al., 2020; Martin and Esser, 2022). However, for some chemical allergens, an unusually frequent T cell activation may significantly contribute to their sensitization potential. Chemical allergen exposure could activate cross-reactive memory T cells in the skin or in draining lymph nodes upon chemical allergen exposure. This scenario is difficult to investigate since T cells lack somatic hypermutations that would allow tracking of early cross-reactive adaptive immune responses (Giesecke et al., 2018). Interestingly, the activation of pre-existing heterologous skin-resident memory T cells is currently explored in cancer therapy, illustrating the potent *in situ* effector functions of this T cell subset (Rosato et al., 2019). T cell activation represents the final key event (key event 4) in the adverse outcome pathway (AOP) of skin sensitization of the OECD. Thus, the further development of alternative *in vitro* T cell-based assay has not only diagnostic but also regulatory potential. So far, integrated testing strategies (ITS), exemplified by the new OECD guideline 429 on “Defined approaches on skin sensitization”, do not comprise T cell activation.

AIM assays offer great potential to contribute to the investigation of chemical-specific T cell responses. Since the efficient *in vitro* generation of chemical allergen-induced T cell epitopes remains the major bottleneck in the development of T cell assays, faster methods could fundamentally accelerate the optimization of assay conditions. One major advantage of AIM assays is their shorter incubation time compared with proliferation-based methods such as the lymphocyte transformation tests (LTT) or amplified T cell libraries (Geiger et al., 2009). In addition, AIM assays are not restricted to the detection of cytokine-producing or proliferating T cell subpopulations but offer the opportunity to follow proliferation patterns and profile cytokine secretion of defined antigen-specific T cell populations. AIM assays are quantitative and compatible with large input cell numbers, e.g., if combined with magnetic enrichment (Bacher and Scheffold, 2015; Reiss et al., 2017; Elias et al., 2020; Saggau et al., 2021). The combination with multi-parameter flow cytometry allows to simultaneously track *in vivo* relevant phenotypic and functional markers in human allergies and the isolation of living T cells for downstream experiments, including TCR HTS (Aparicio-Soto et al., 2020). Besides CD154 and CD137, other activation markers used alone or in combination have been discussed including CD134 (OX40), CD25, CD69, CD71 or HLA-DR (Bacher and Scheffold, 2013). AIM assays are currently extensively used to characterize SARS-CoV-2-specific T cell responses (Bacher et al., 2020; Jung et al., 2021; Tarke et al., 2021). However, for each activation marker and chemical allergen, careful validation to prove TCR-mediated activation, e.g., antigen-specific restimulation of T cell clones, is required. Furthermore, high background expression, e.g., of CD137, CD25 or CD69, on T cells from some buffy coats, or slow expression kinetics can impede the detection or quantification of rare antigen-specific T cells, respectively.

Taken together, the exploration of frequencies and TCR repertoires of chemical allergen-specific T cell subpopulations can significantly advance our understanding of chemical sensitization pathomechanisms (Villani et al., 2021). Methodological advances in protein antigen-specific T cell assessment may be adapted for the detection of chemical-specific T cells, as shown in this proof-of-principle study with the model allergen TNBS. AIM assays could speed up the development of T cell-based alternative diagnostic and predictive *in vitro* tests. This could pave the way for the inclusion of T cell responses in the emerging era of next-generation risk assessment.

## Data Availability

The TCR sequencing datasets presented in this study can be found online European Nucleotide Archive (ENA), accession number PRJEB49381.

## Abbreviations

AIM, activation-induced marker; APC, antigen-presenting cells; CDR, complementarity determining region; CFSE, carboxyfluorescein diacetate succinimidyl ester; HTS, high-throughput sequencing; MHC, major histocompatibility complex; PBMC, peripheral blood mononuclear cells; PMA/iono, phorbol myristate acetate/ionomycin; TCR, T cell receptors; TNBS, 2,4,6-trinitrobenzenesulfonic acid; TNP, trinitrophenyl.
