# Supplementary material for: Frequencies and TCR Repertoires of Human 2,4,6-Trinitrobenzenesulfonic Acid-specific T Cells
Source: Front Toxicol. 2022 Feb 22;4:827109. doi: 10.3389/ftox.2022.827109 (PMC8915883; doi:10.3389/ftox.2022.827109)
Supplement: Supplementary file 1 [file Table1.docx]

Supplementary Material

**Table S1**. **Sample overview.** This table lists buffy coats, frequency percentages of activated T cells and CD69 co-expression (raw values from **Figures 1, S6**) and unique buffy coat identifiers (donor symbols). Shaded areas indicate samples from which T cells were sorted for TCR HTS. Empty values indicate that samples have not been analyzed. Bold and underlined values indicate samples, where T cells have been sorted for *in vitro* expansion and restimulation experiments.

| **Buffy coat** | **CD4+ memory T cells** (5 hour assays) | | | | | **CD8+ memory T cells** (16 hour assays) | | | | | **Symbol** |
| --- | --- | --- | --- | --- | --- | --- | --- | --- | --- | --- | --- |
|  | **Well size** | **Control** | | **TNBS** | | **Well size** | **Control** | | **TNBS** | |  |
|  |  | **% CD154+** | **% CD69**  **co-exp.^**^** | **% CD154+** | **% CD69**  **co-exp.^**^** |  | **% CD137+** | **% CD69 co-exp.^**^** | **% CD137+** | **% CD69**  **co-exp.^**^** |  |
| ABAB3 |  |  |  |  |  | 6-well | 0.11 | 78.38 | 0.27 | 81.58 | 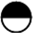 |
| CACB2 | 6-well^*&^ | 0.01 |  | 0.04 |  |  |  |  |  |  | 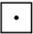 |
| **CACB3** | 6-well | 0.07 |  | **0.11** |  |  |  |  |  |  | 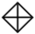 |
| HOEB38 | 12-well^*&^ | 0.02 |  | 0.07 |  |  |  |  |  |  | 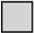 |
| IWB1 | 12-well | 0.01 | 43.8 | 0.02 | 68.2 |  |  |  |  |  | 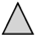 |
| IWB2 | 12-well | 0.01 | 100.0 | 0.02 | 100.0 |  |  |  |  |  | 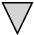 |
| **LMB1** | 12-well | 0.04 | 76.9 | 0.09 | 76.7 | 12-well | 1.19 | 43.88 | **5.25** | 69.8 | 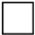 |
| **MASB7** |  |  |  |  |  | 12-well | 0.37 | 46.79 | **0.84** | 75.4 | 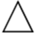 |
| **MLB20** | 12-well | 0.10 | 52.4 | **0.25** | 95.3 | 12-well | 0.49 | 73.3 | 0.62 | 76.3 | 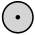 |
| MLB21 | 12-well | 0.05 | 50.0 | 0.08 | 83.9 |  |  |  |  |  | 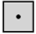 |
| MLB23 | 6-well*^&^ | 0.17 |  | 0.18 |  |  |  |  |  |  | 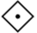 |
| MLB24 | 6-well | 0.00 |  | 0.03 |  | 96-well | 0.07 | 25.0 | 0.90 | 28.3 | 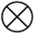 |
| MLB27 |  |  |  |  |  | 12-well^*^ | 0.18 | 39.24 | 1.76 | 59.33 | 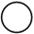 |
| MLB31 | 12-well | 0.04 | 43.33 | 0.06 | 65.95 | 12-well | 0.47 | 16.3 | 1.23 | 62.8 | 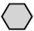 |
| MLB35 |  |  |  |  |  | 12-well | 0.37 |  | 0.89 |  | 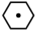 |
| **MLB36** |  |  |  |  |  | 12-well | 0.03 | 77.1 | **0.13** | 95.4 | 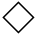 |
| MLB37 |  |  |  |  |  | 12-well | 0.08 | 96.3 | 0.42 | 99.6 | 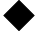 |
| MLB38 |  |  |  |  |  | 12-well | 0.12 | 21.4 | 1.27 | 49.6 | 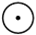 |
| MLB39 |  |  |  |  |  | 12-well | 0.49 | 20.5 | 1.13 | 34.2 | 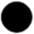 |
| MLB40 |  |  |  |  |  | 12-well | 0.26 |  | 0.19 |  | 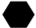 |
| **MLB42a** |  |  |  |  |  | 12-well | 0.07 | 38.46 | **0.11** | 55.3 | 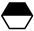 |
| MLB43 |  |  |  |  |  | 12-well | 0.29 | 56.2 | 0.12 | 41.2 | 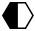 |
| MLB44 |  |  |  |  |  | 12-well | 0.10 | 38.5 | 0.10 | 54.3 | 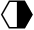 |
| MLB45 |  |  |  |  |  | 12-well | 0.18 | 93.3 | 0.22 | 86.2 | 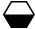 |
| **Means ± SD** | | 0.05 ± 0.05 | 61.1 ± 20.7 | 0.09 ± 0.07 | 81.7 ± 12.8 |  | 0.29 ± 0.27 | 58.9 ± 22.8 | 0.91 ± 1.19 | 67.7 ± 19.0 |  |
| **Background (control) corrected^#^** | | | | **0.04 ± 0.02** | **20.6 ± 7.90** |  | | | **0.62 ± 0.92** | **8.80 ± 3.80** |  |

^*^control samples analyzed on 24-well plates. ^&^TNBS samples analyzed on 6-well plates. ^**^percentage of CD154+ or CD137+ T cells that co-express CD69. ^#^Values from the respective controls were subtracted.
