# Supplementary material for: Frequencies and TCR Repertoires of Human 2,4,6-Trinitrobenzenesulfonic Acid-specific T Cells
Source: Front Toxicol. 2022 Feb 22;4:827109. doi: 10.3389/ftox.2022.827109 (PMC8915883; doi:10.3389/ftox.2022.827109)
Supplement: Supplementary file 2 [file Table2.DOCX]

Supplementary Material

**Table S2. Overview of TNBS-specific T cell clones and lines.** This table lists sorted T cells from CD154/CD137 upregulation assays. For lines, the initially sorted cell number is given in the line designation in bold. After *in vitro* expansion, cells were tested for TNBS specificity in restimulation assays. “+” indicates a positive result, “-“ indicates a negative result. In addition, some clones and lines were tested for MHC restriction.

| **Buffy coat** | **T cell clone/line designation** | **Restimulation result** | **Tested for MHC restriction** |
| --- | --- | --- | --- |
| CACB3 | CD4_5T | + | yes |
| CACB3 | CD4_19T | + | yes |
| CACB3 | CD4_15T | - | no |
| CACB3 | CD4_1T | + | yes |
| CACB3 | CD4_2T | + | yes |
| CACB3 | CD4_14T | + | yes |
| CACB3 | CD4_16T | + | yes |
| CACB3 | CD4_20T | + | yes |
| MLB20 | CD4_T3-**50** | + | no |
| MLB20 | CD4_8D | - | no |
| MLB20 | CD4_11E | + | no |
| MLB20 | CD4_10D | + | no |
| MLB20 | CD4_9B | - | no |
|  |  |  |  |
| LMB1 | CD8_F5 | + | yes |
| LMB1 | CD8_F6 | + | yes |
| LMB1 | CD8_B3-**100** | - | no |
| MASB7 | CD8_12D2 | + | yes |
| MASB7 | CD8_9C1 | - | no |
| MASB7 | CD8_4A2 | - | no |
| MLB42a | CD8_1A-**50** | + | no |
| MLB42a | CD8_1B-**50** | - | no |
| MLB42a | CD8_1C-**50*** | + | no |
| MLB42a | CD8_1D-**50** | + | yes |
| MLB42a | CD8_4D | + | yes |
| MLB42a | CD8_4E | + | yes |
| MLB42a | CD8_4F | + | yes |
| MLB36 | CD8_B7 | + | no |
| MLB36 | CD8_8E | + | no |

*analyzed by TCR-HTS (see Table S3)
