# Supplementary material for: Frequencies and TCR Repertoires of Human 2,4,6-Trinitrobenzenesulfonic Acid-specific T Cells
Source: Front Toxicol. 2022 Feb 22;4:827109. doi: 10.3389/ftox.2022.827109 (PMC8915883; doi:10.3389/ftox.2022.827109)
Supplement: Supplementary file 3 [file DataSheet1.pdf]

Figure S1

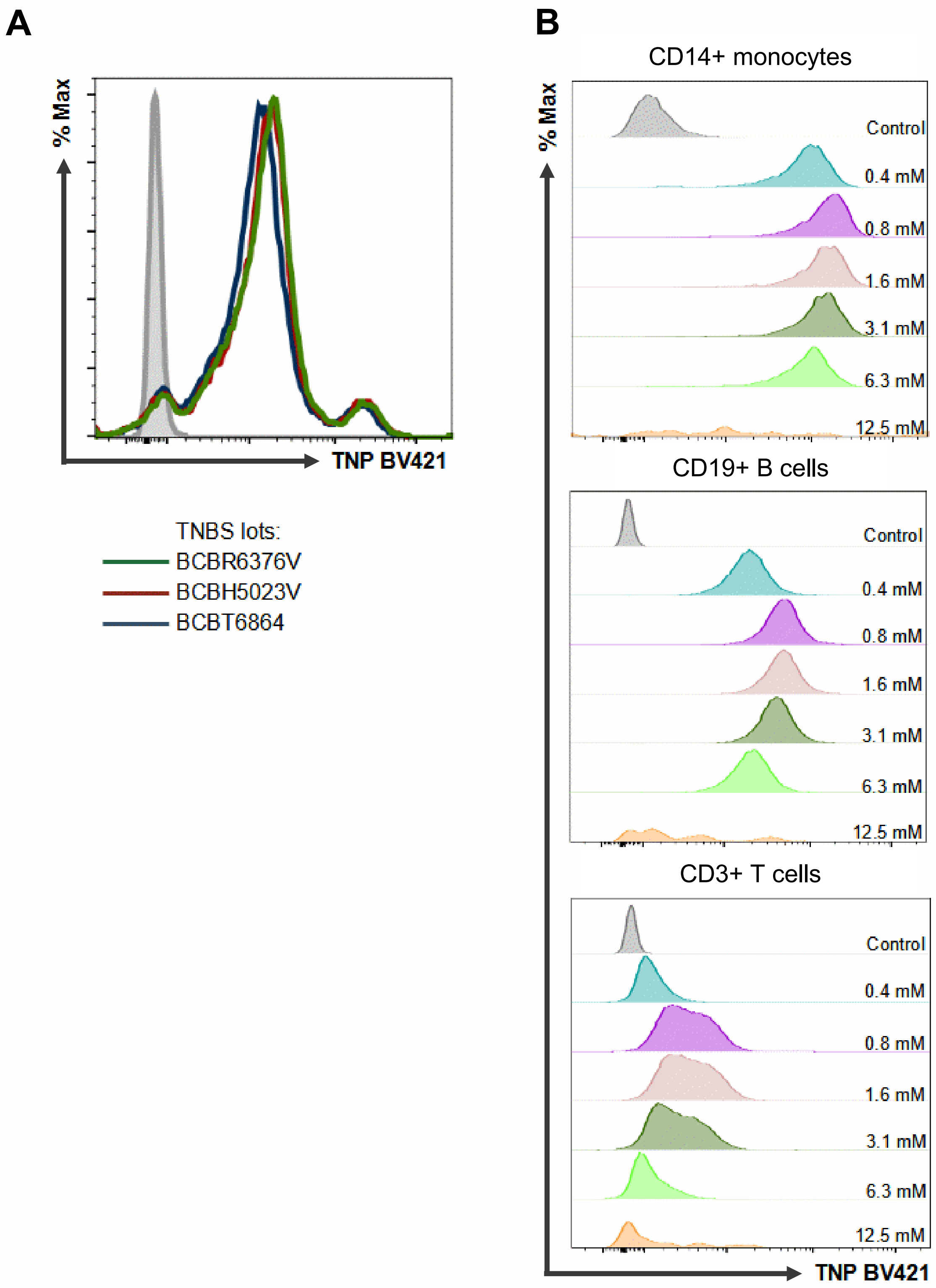

Figure S1. TNBS modification of PBMC. (**A**) Reproducibility of TNBS-modifications. PBMC were treated with 3 mM TNBS from different lots, stained with anti-TNP antibody and analyzed by flow cytometry. Gated on live cells. (**B**) TNBS modification of cell subtypes. PBMC were treated with the indicated TNBS concentrations in a two-fold dilution series starting from 12.5 mM TNBS. TNBS-modification of cell subpopulations were analyzed according to the indicated surface markers after live cell gating. For both graphs, one representative experiment is shown from n = 2 – 3 experiments.

Figure S2

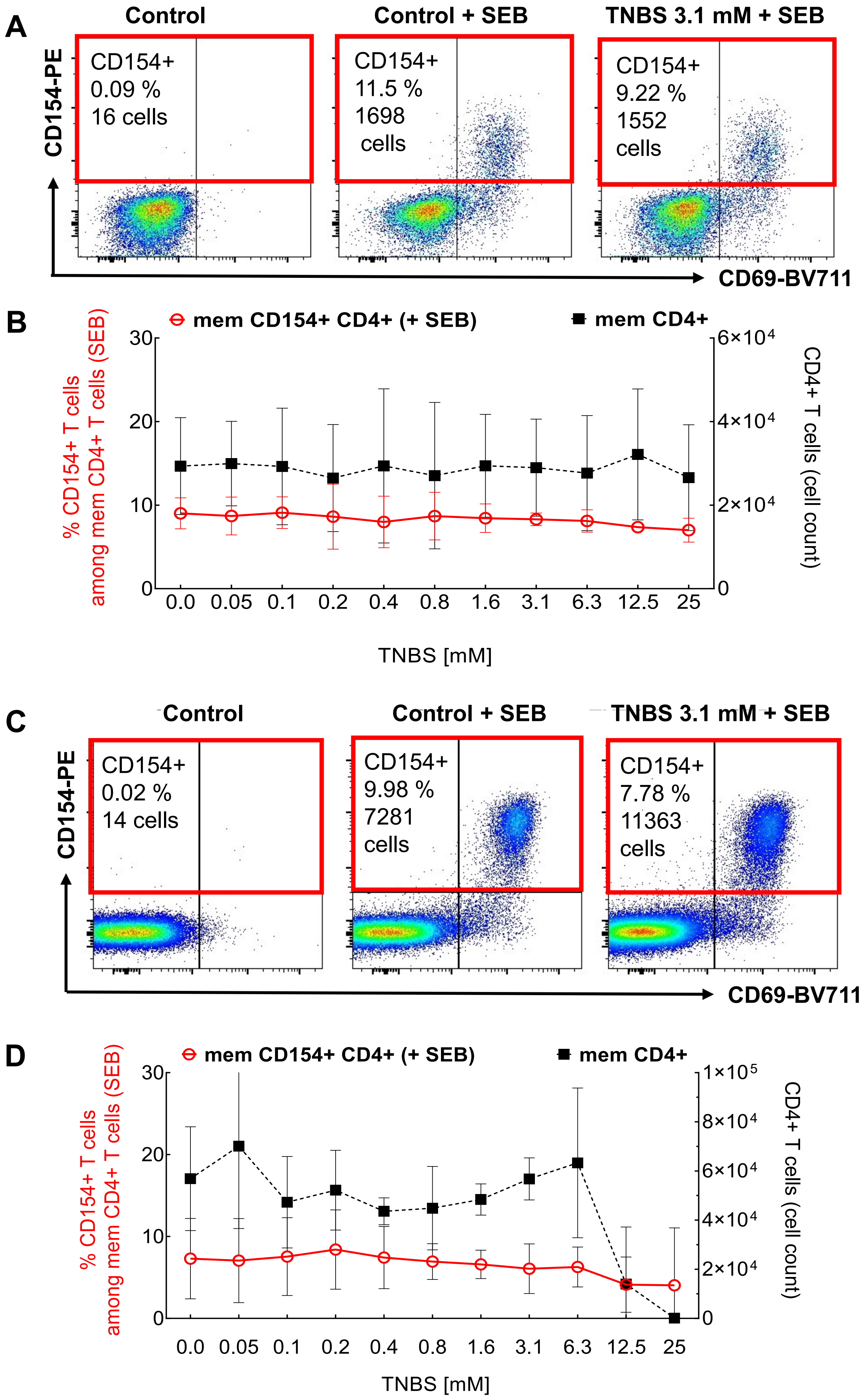
A
Control
Control + SEB
TNBS 3.1 mM + SEB
CD154-PE
CD154+
0.09 %
16 cells
CD154+
11.5 %
1698
cells
CD154+
9.22 %
1552
cells
CD69-BV711
B
mem CD154+ CD4+ (+ SEB)
mem CD4+
% CD154+ T cells
among mem CD4+ T cells (SEB)
CD4+ T cells (cell count)
TNBS [mM]
0.0
0.05
0.1
0.2
0.4
0.8
1.6
3.1
6.3
12.5
25
0
10
20
30
2×10^4
4×10^4
6×10^4
C
Control
Control + SEB
TNBS 3.1 mM + SEB
CD154-PE
CD154+
0.02 %
14 cells
CD154+
9.98 %
7281
cells
CD154+
7.78 %
11363
cells
CD69-BV711
D
mem CD154+ CD4+ (+ SEB)
mem CD4+
% CD154+ T cells
among mem CD4+ T cells (SEB)
CD4+ T cells (cell count)
TNBS [mM]
0.0
0.05
0.1
0.2
0.4
0.8
1.6
3.1
6.3
12.5
25
0
10
20
30
2×10^4
4×10^4
6×10^4
8×10^4
1×10^5

Figure S2. Effects of TNBS on viability and T cell CD154 upregulation. **(A, B)** Analysis of "responder" CD4+ memory T cells in a CD154 upregulation assay (5 h). PBMC were CFSE labeled and modified with PBS (control) or TNBS (0.05 – 25 mM, two-fold dilution series) and mixed as APCs in a 1:1 ratio with unmodified "responder" PBMC. In addition, some samples were stimulated with SEB, a superantigen activating a large fraction of T cells. **(A)** Representative dot plots showing CD154 expression upon different stimulation conditions (red boxes, MLB19). **(B)** Graph summarizing data from n ≥ 3 experiments with different buffy coats showing frequencies of SEB-stimulated CD154+CD4+ memory T cells (left y-axis) and total CD4+ memory T cell numbers (right y-axis) in the presence of APC treated with different TNBS concentrations. **(C, D)** Analysis of T cells from TNBS-treated PBMC (APC, without CFSE label and "responder" cells, thus with double input cell number to maintain optimal cell surface densities). **(C)** Representative dot plots (as in (A), MLB16). **(D)** Graph summarizing data from n ≥ 3 experiments, as in (B). All data were gated on live, CD3+, single, CD4+ memory T cells (see **Figure S4**) and have been performed on a 96-well plate. Lines represent mean values, error bars the standard deviation.

Figure S3

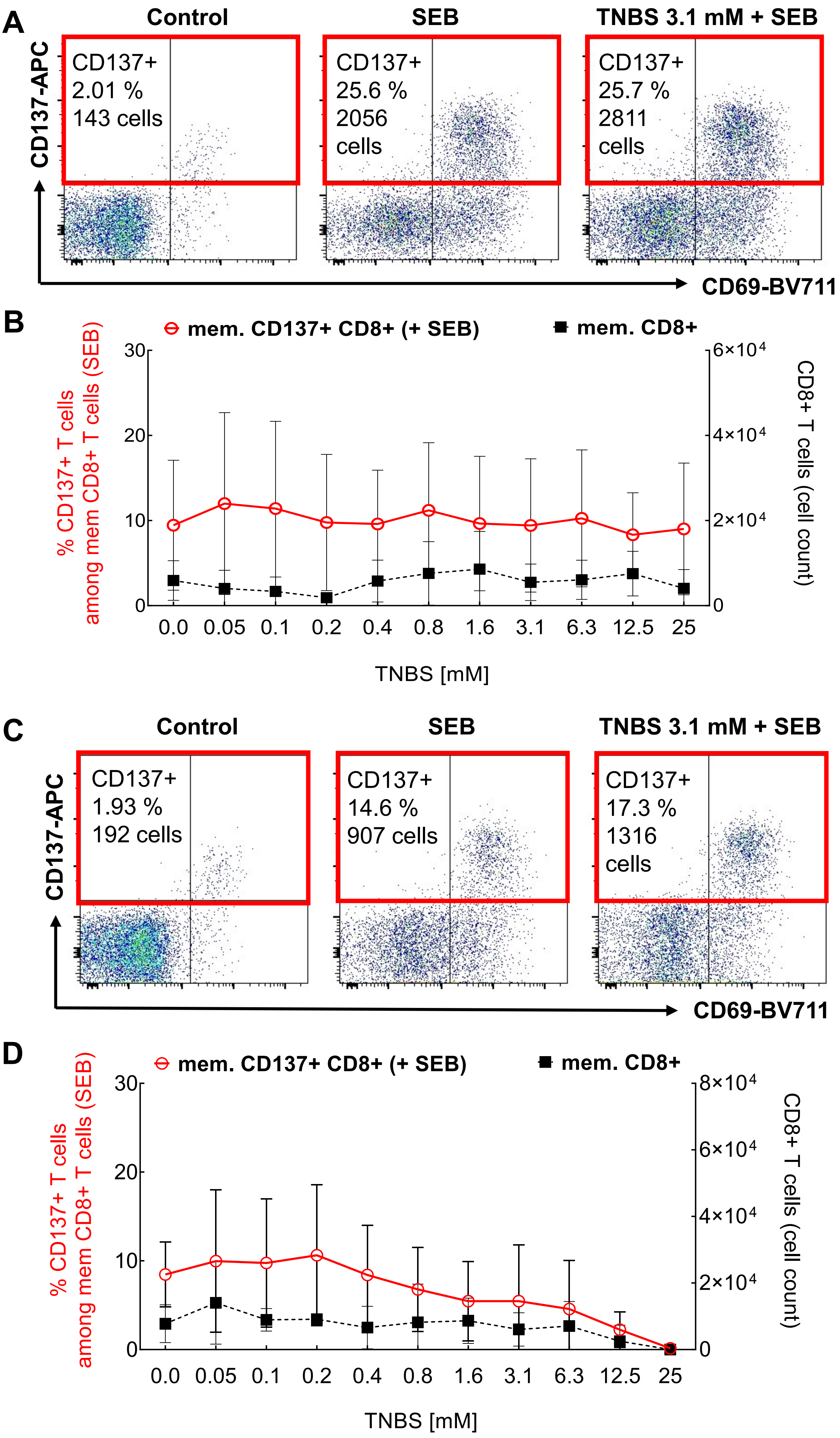

A
Control
SEB
TNBS 3.1 mM + SEB
CD137-APC
CD137+
2.01 %
143 cells
CD137+
25.6 %
2056
cells
CD137+
25.7 %
2811
cells
CD69-BV711
B
mem. CD137+ CD8+ (+ SEB)
mem. CD8+
% CD137+ T cells
among mem CD8+ T cells (SEB)
CD8+ T cells (cell count)
0
10
20
30
0
2×10⁴
4×10⁴
6×10⁴
0.0
0.05
0.1
0.2
0.4
0.8
1.6
3.1
6.3
12.5
25
TNBS [mM]
C
Control
SEB
TNBS 3.1 mM + SEB
CD137-APC
CD137+
1.93 %
192 cells
CD137+
14.6 %
907 cells
CD137+
17.3 %
1316
cells
CD69-BV711
D
mem. CD137+ CD8+ (+ SEB)
mem. CD8+
% CD137+ T cells
among mem CD8+ T cells (SEB)
CD8+ T cells (cell count)
0
10
20
30
0
2×10⁴
4×10⁴
6×10⁴
8×10⁴
0.0
0.05
0.1
0.2
0.4
0.8
1.6
3.1
6.3
12.5
25
TNBS [mM]

Figure S3. Effects of TNBS on viability and T cell CD137 upregulation. **(A, B)** Analysis of "responder" CD8+ memory T cells in a CD137 upregulation assay (16 h). PBMC were CFSE labeled and modified with PBS (control) or TNBS (0.05 – 25 mM, two-fold dilution series) and mixed as APCs in a 1:1 ratio with unmodified "responder" PBMC. In addition, some samples were stimulated with SEB. **(A)** Representative dot plots showing CD137 expression upon different stimulation conditions (red boxes, MASB7). **(B)** Graph summarizing data from n ≥ 3 experiments with different buffy coats and TNBS concentrations for APC modification showing frequencies of SEB-stimulated CD137+CD8+ memory T cells (left y-axis) and total CD8+ memory T cell numbers (right y-axis). **(C, D)** Analysis of T cells from TNBS-treated PBMC (APC). **(C)** Representative dot plots (as in (A), MASB7). **(D)** Graph summarizing data from n ≥ 2-3 experiments, as in (B). All data were gated on live, CD3+, single, CD8+ memory T cells (see **Figure S4**) and have been performed on a 96-well plate. Lines represent mean values, error bars the standard deviation.

Figure S4

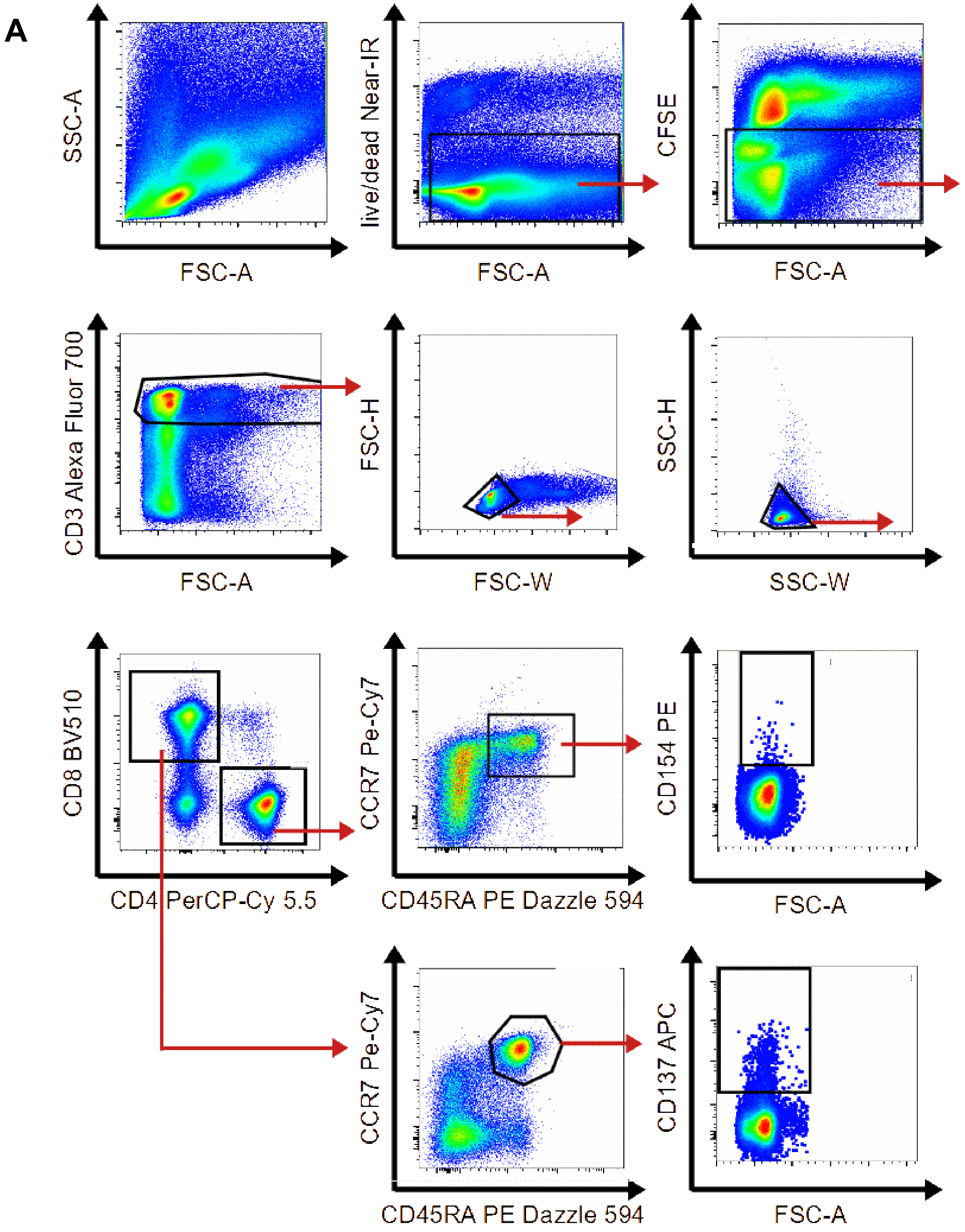

Figure S4. Gating strategy. Responder (non-modified) PBMC were gated on live, CFSE- (excluding CFSE-high population), CD3+, single, CD4+ or CD8+, naïve (CCR7+, CD45RA+) or memory (non-naïve) T cells to analyze TNBS-induced CD154 (5 h) and CD137 (16 h) expression (example from donor MLB20). Expression of further activation markers, e.g. CD69, was assessed among total and antigen-specific CD154+ and CD137+ T cells.

Figure S5

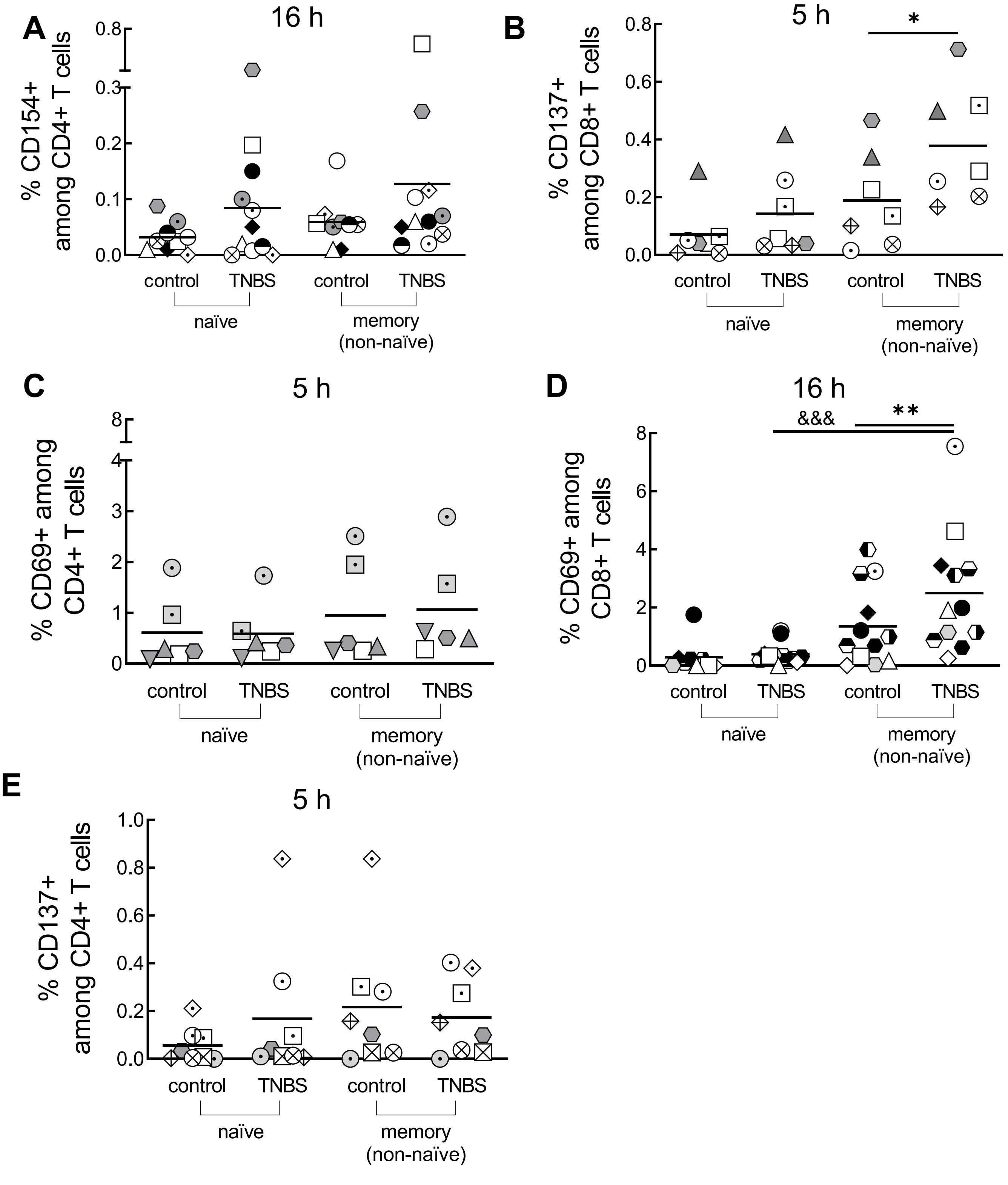

Figure S5. Additional activation marker expression in CD154/CD137 upregulation assays. PBMC were CFSE-labeled and modified with PBS (control) or TNBS as APC and incubated in a 1:1 ratio with unmodified "responder" PBMC for the indicated assay times. **(A)** CD154 upregulation assay (n = 11 buffy coats). (**B)** CD137 upregulation assay (n = 7 buffy coats). **(C, D)** CD69 upregulation by CD4+ **(C)** or CD8+ **(D)** T cells. **(E)** CD137 upregulation by CD4+ T cells. Symbols represent buffy coat identifiers (**Table S1**). Horizontal lines indicate the mean values. Statistical significances were determined by non-parametric Mann-Whitney t-test (P<.*0.5, **0.01 vs memory control; &&& P<.001 vs naïve TNBS).

□ LMB1, ⬡ ML_B31

Figure S6

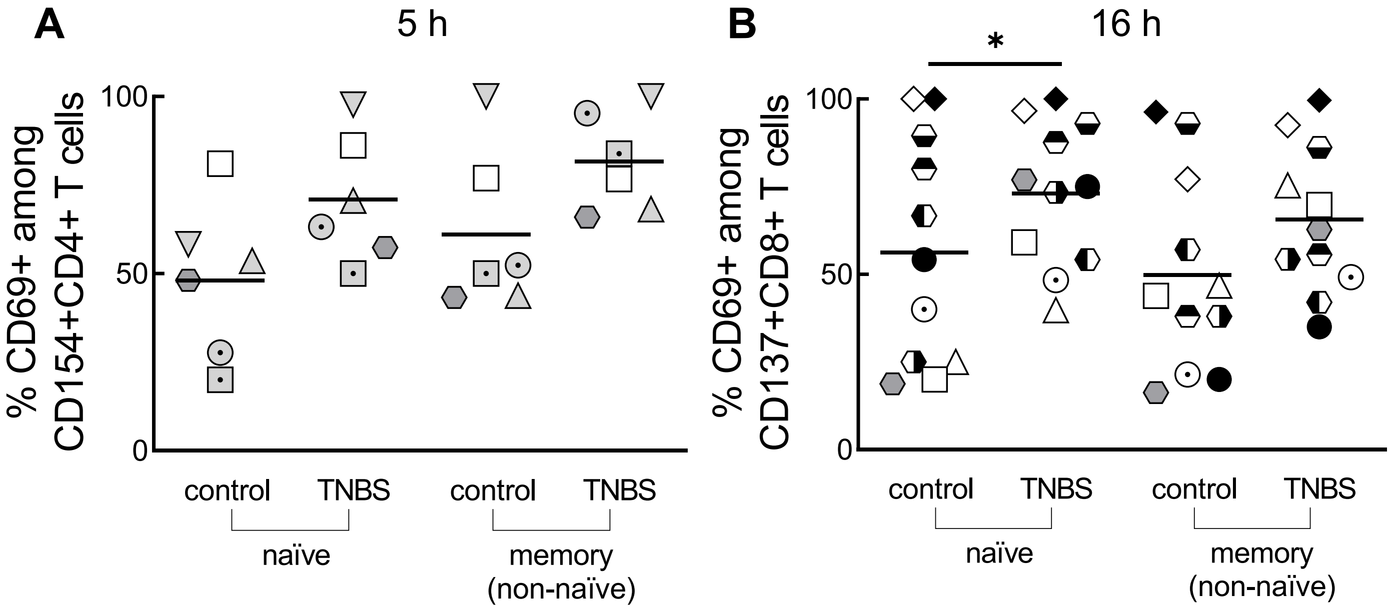

Figure S6. CD69 co-expression analysis. PBMC were CFSE-labeled and modified with PBS (control) or TNBS as APC and incubated in a 1:1 ratio with unmodified "responder" PBMC for the indicated times. **(A)** CD69 co-expression by CD154+CD4+ T cells. **(B)** CD69 co-expression by CD137+CD8+ T cells. Symbols represent buffy coat identifiers (**Table S1**). Horizontal lines indicate the mean values. Statistical significances were determined by non-parametric Mann-Whitney t-test (P<.*0.5 vs memory control).

Figure S7

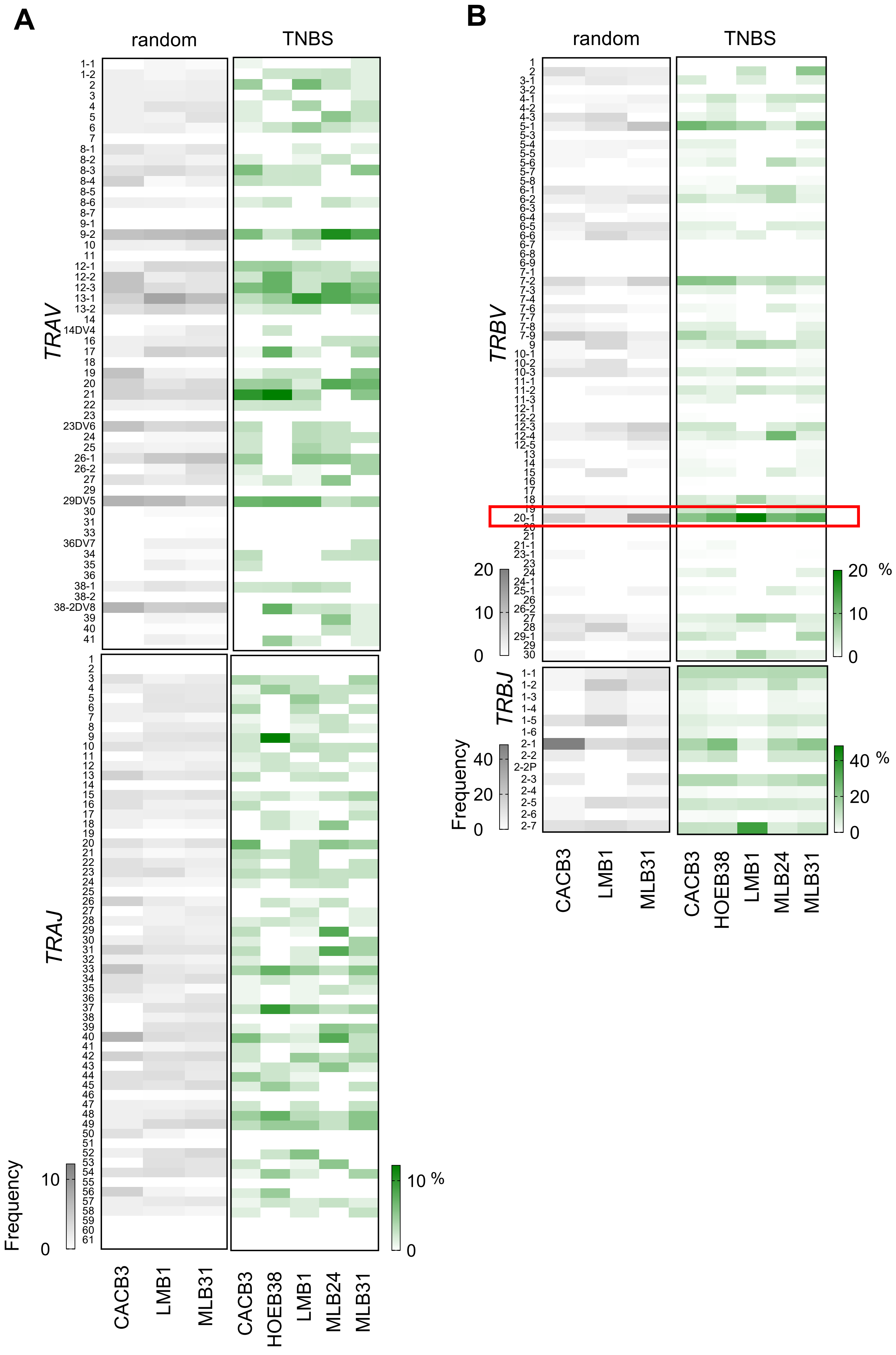
A
random
TNBS
TRAV
TRAJ
Frequency
10
0
10 %
0
CACB3
LMB1
MLB31
CACB3
HOEB38
LMB1
MLB24
MLB31
B
random
TNBS
TRBV
TRBJ
20
10
0
20 %
10
0
Frequency
40
20
0
40 %
20
0
CACB3
LMB1
MLB31
CACB3
HOEB38
LMB1
MLB24
MLB31

Figure S7, cont.

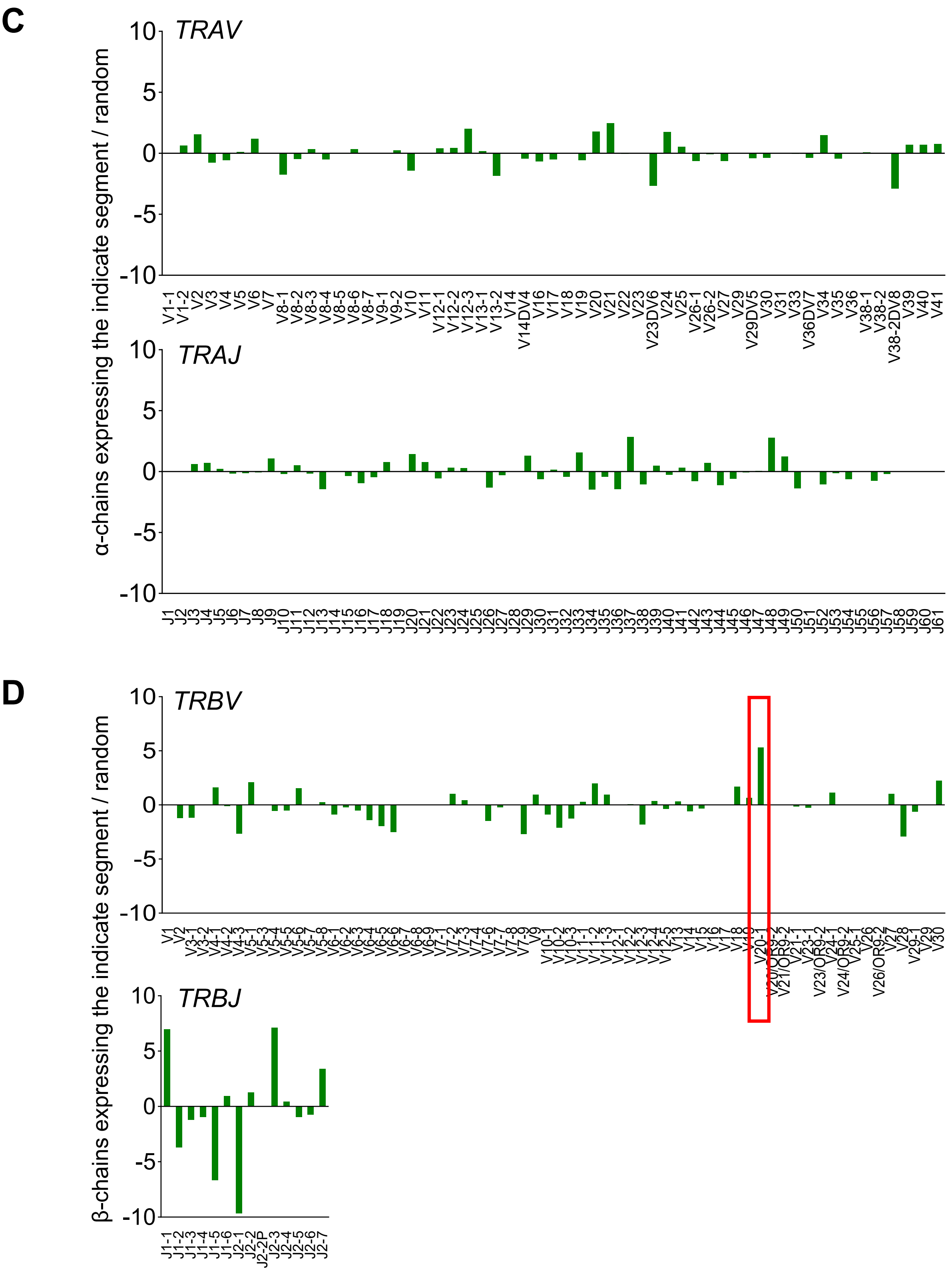

Figure S7, cont.

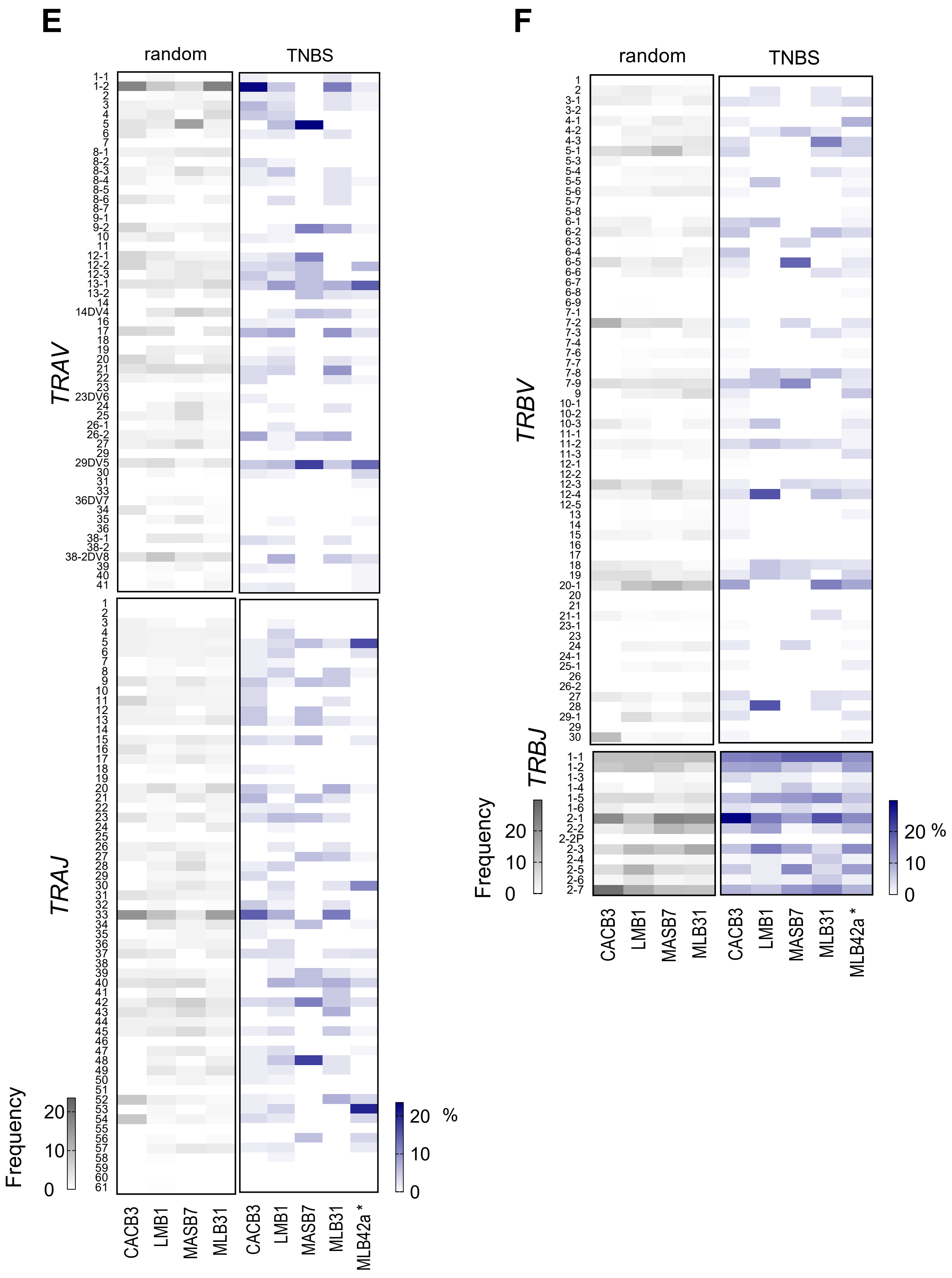

Figure S7, cont.

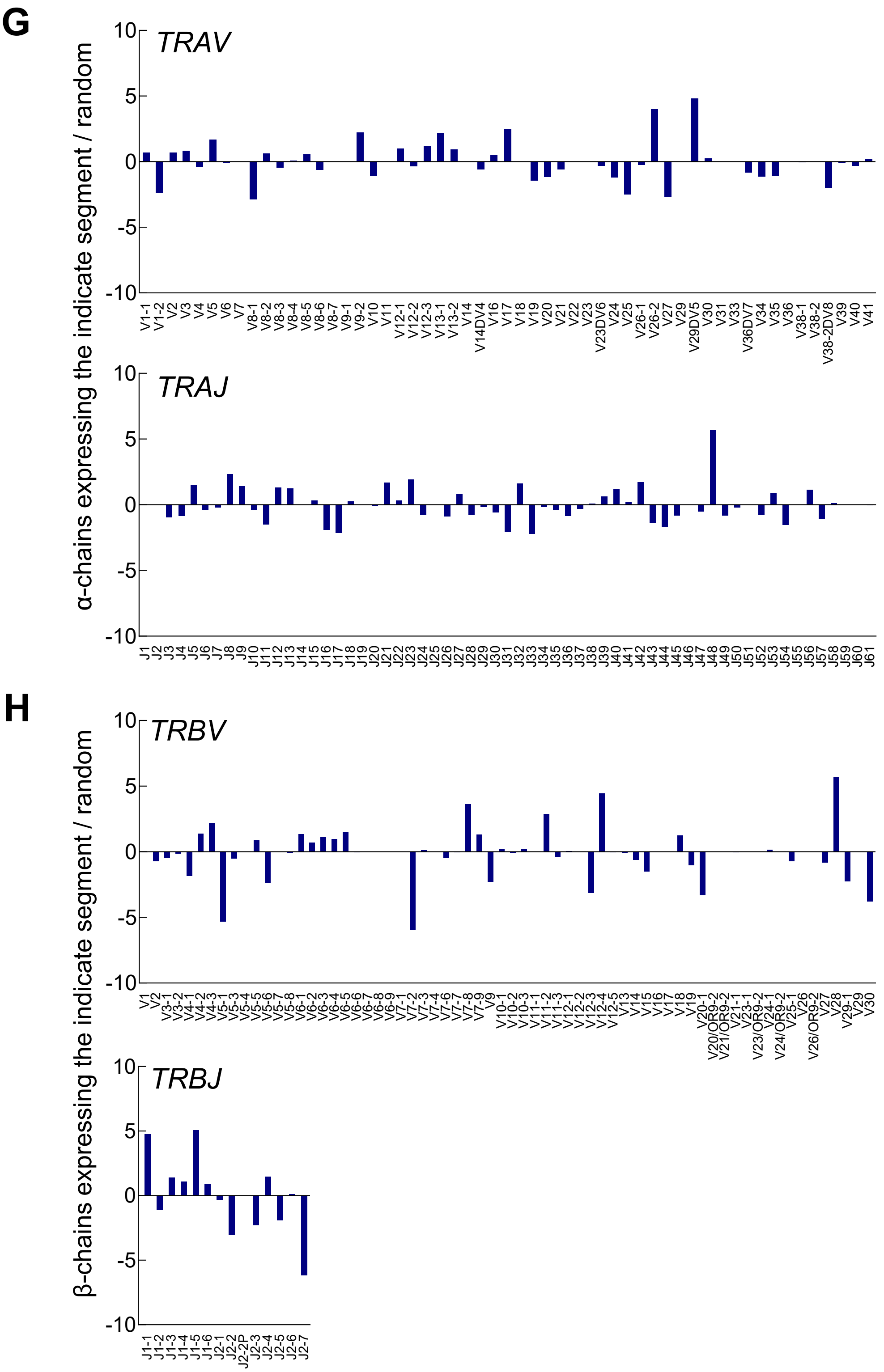

Figure S7. Gene segment use by TNBS-specific T cells. TNBS-specific CD154+CD4+ and CD137+CD8+ memory T cells were sorted from 5 h and 16 h experiments, respectively, and their TCR were sequenced (**Table S3**). Random CD4+ and CD8+ memory T cells from the same buffy coats served as control. Heatmaps and bar charts depict TCR α- **(A, C, E, G)** and β-chain **(B, D, F, H)** V- and J-segment gene use expressed as TCR diversity frequencies. Random TCR are shown in grey, TNBS-specific TCR in green (CD4+ T cells) and blue (CD8+ T cells). Highlighted in red **(B, D)** is the TRBV20-1 segment. Statistical significance was determined by multiple t-test with Sidak-Holm correction for multiple comparison (n = 3 – 5, adj.-P-value = 0.004).

Figure S8

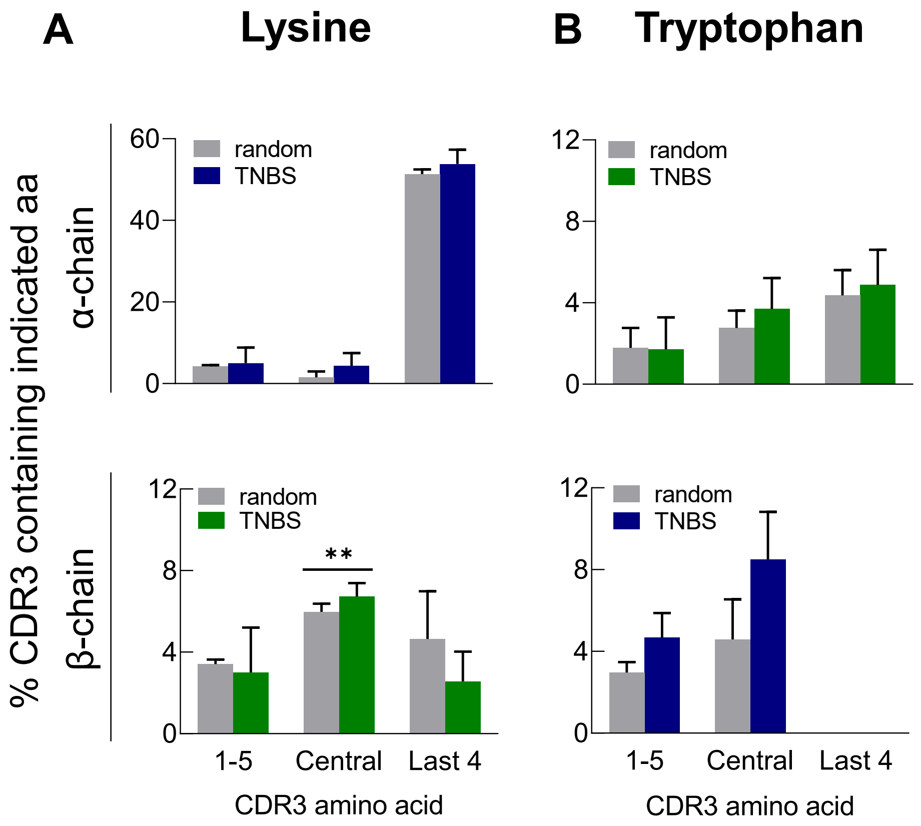

Figure S8. Lysine and tryptophan location in the CDR3. Random and TNBS-specific CD4+ and CD8+ memory T cells were sorted and their TCR sequenced (**Table S3**). (**A, B**) The graphs depict lysine (**A**) and tryptophan (**B**) occurrence according to their locations in the CDR3 of TCR α- (upper panels) and *β*-chains (lower panels) among random (grey) and TNBS-specific CD4+ (green) and CD8+ (blue) T cells (see also **Figure 3G**). Statistical significance was determined by t-test and corrected for multiple comparisons with the Holm-Sidak's method (n = 3 – 5, **P<.01).

Figure S9

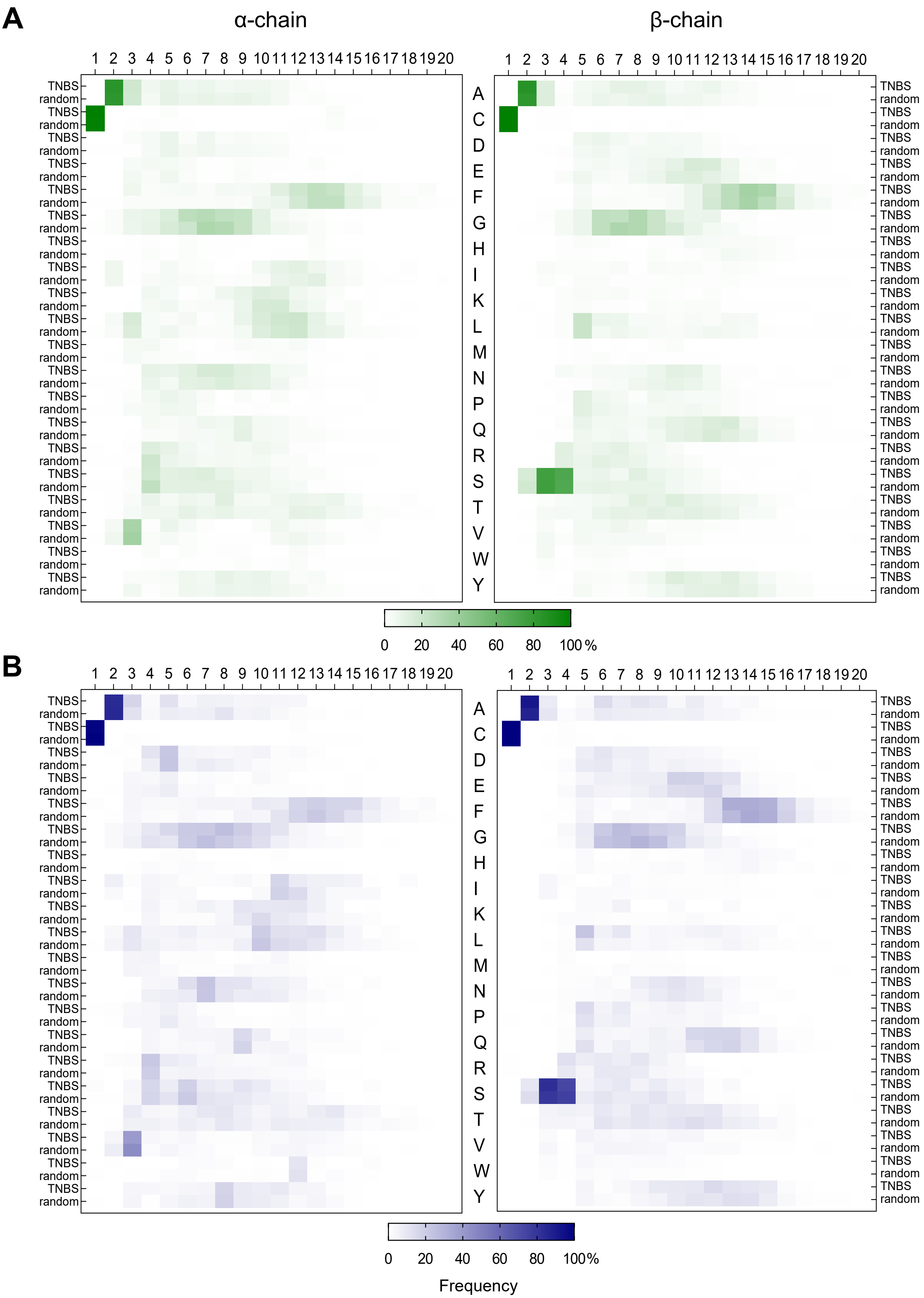
A
α-chain
β-chain
1 2 3 4 5 6 7 8 9 10 11 12 13 14 15 16 17 18 19 20
TNBS
random
A C D E F G H I K L M N P Q R S T V W Y
0 20 40 60 80 100 %
B
α-chain
β-chain
0 20 40 60 80 100%
Frequency

Figure S9, cont.

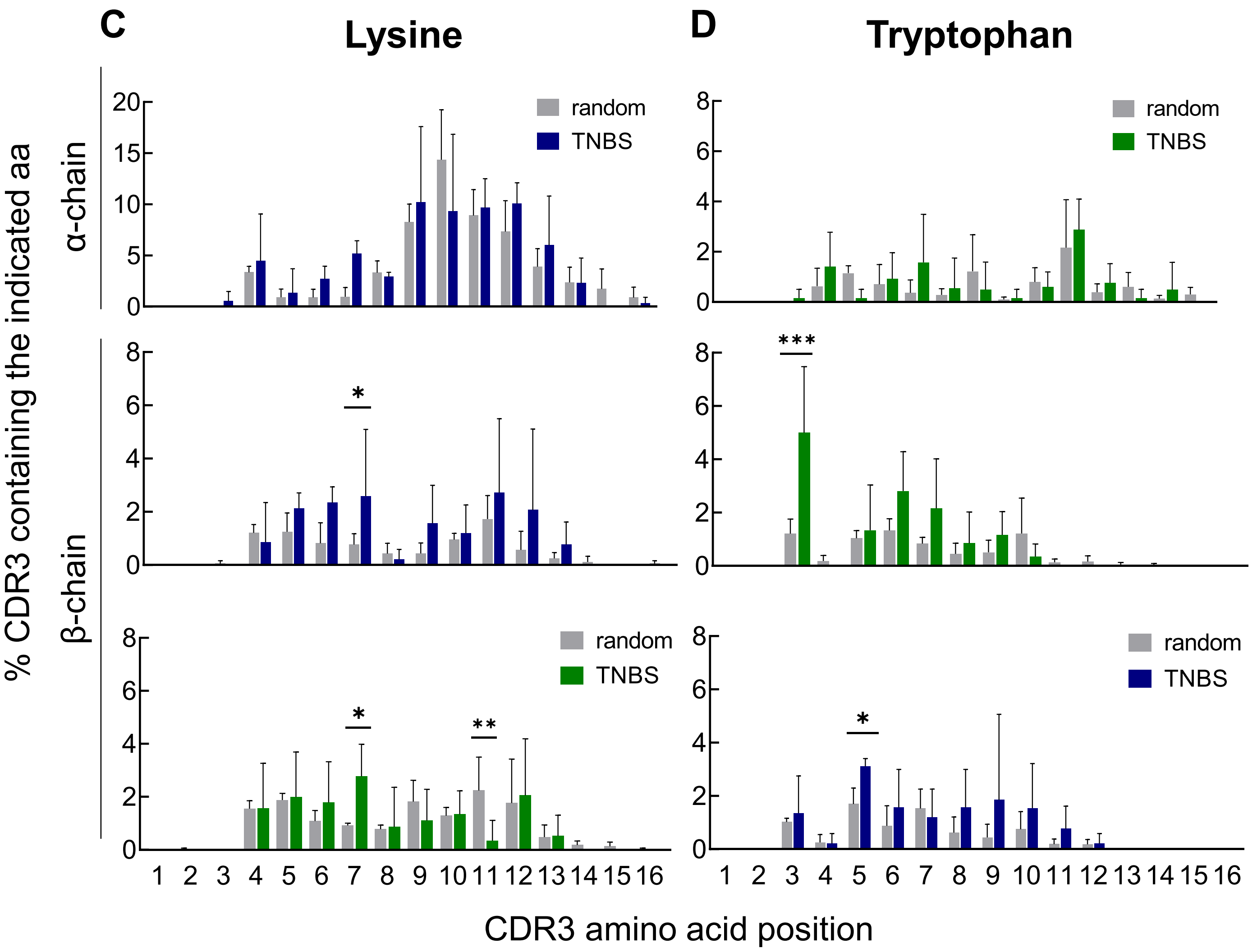

Figure S9. Positional exact analysis of CDR3 amino acids. Random and TNBS-specific CD4+ and CD8+ memory T cells were sorted and their TCR sequenced (**Table S3**). (**A, B**) Heatmaps depicting the occurrence of all amino acids in the CDR3 according to their positions (positions 1 to 16 cover most of the CDR3 length) for TCR α- and β-chains of CD4+ (green, **A**) and CD8+ (blue, **B**) T cells (mean values, TCR diversity, n = 3-5). (**C, D**) Bar charts depict lysine (**C**) and tryptophan (**D**) occurrence according to their CDR3 position in TCR α- (upper panels) and *β*-chains (middle and lower panels) for TCR from random (grey) and TNBS-specific CD4+ (green) and CD8+ (blue) T cells. Statistical significances were determined by multiple t-test and corrected for multiple comparison with the Sidak-Holm's method (n = 3 – 5, P<*0.05,**0.01, ***0.001).
