# Supplementary material for: Frequencies and TCR Repertoires of Human 2,4,6-Trinitrobenzenesulfonic Acid-specific T Cells
Source: Front Toxicol. 2022 Feb 22;4:827109. doi: 10.3389/ftox.2022.827109 (PMC8915883; doi:10.3389/ftox.2022.827109)
Supplement: Supplementary file 4 [file Table3.DOCX]

Supplementary Material

**Table S3**. **Overview of TCR sequencing data.** This table lists the number of sorted cells from the respective buffy coats, obtained TCR sequences (counts, each count is represented by one UMI) and TCR diversity (clonotypes).

| Buffy coat | CD4+ memory T cells* | | | | | | CD8+ memory T cells** | | | | | | |
| --- | --- | --- | --- | --- | --- | --- | --- | --- | --- | --- | --- | --- | --- |
|  | Control (random T cells) | | | TNBS-specific (CD154+ ) | | | Control (random T cells) | | | | TNBS-specific (CD137+) | | |
|  | Sorted cells | Counts /  diversity | | Sorted cells | Counts /  diversity | | Sorted cells | Counts /  diversity | | Sorted cells | | Counts /  diversity | |
|  |  | α | β |  | α | β |  | α | β |  | | α | β |
| CACB3 | 500 | 91  69 | 144  109 | 393 | 296  129 | 197  116 | 500 | 79  50 | 169  85 | 450 | | 100  61 | 122  60 |
| HOEB38 |  |  |  | 524 | 71  41 | 77  49 |  |  |  |  | |  |  |
| LMB1 | 10 000 | 1524  1159 | 1765  1396 | 600 | 208  122 | 178  114 | 10 000 | 820  453 | 1345  552 | 2 556 | | 157  98 | 242  155 |
| MLB24 |  |  |  | 503 | 66  37 | 88  49 |  |  |  |  | |  |  |
| MLB31 | 10 000 | 1015  708 | 1425  976 | 369 | 111  72 | 62  45 | 10 000 | 607  345 | 867  518 | 727 | | 73  41 | 59  36 |
| MLB42a ^§^ |  |  |  |  |  |  |  |  |  | 5 000 | | 32471  108 | 13225  22 |

*Sorted from 5 hour assays, **sorted from 16 h assays; ^§^CD137^+^CD8^+^ T cell line (MLB42a_CD8_1C-50, consisting of initially 50 sorted cells that had been expanded *in vitro*, see also **Table S2**)
