# Supplementary material for: Frequencies and TCR Repertoires of Human 2,4,6-Trinitrobenzenesulfonic Acid-specific T Cells
Source: Front Toxicol. 2022 Feb 22;4:827109. doi: 10.3389/ftox.2022.827109 (PMC8915883; doi:10.3389/ftox.2022.827109)
Supplement: Supplementary file 5 [file Table4.DOCX]

***Supplementary Material***

# Supplementary Methods

## Blood samples and isolation of PBMC

T cell media (TCM) consisted of RPMI 1640 medium supplemented with 5% (v/v) human AB serum (both PAN Biotech), 2 mM glutamine (GlutaMAX), 55 µM β-mercaptoethanol (both Thermo Fisher Scientific), 1x non-essential amino acids, 1 mM sodium pyruvate, 10 mM HEPES (all PAN Biotech), 1x Penicillin/Streptomycin (Capricorn Scientific) and 1.5 mM CaCl_2_ (Sigma Aldrich) for optimal T cell responses (Zimmermann et al., 2015)

## Antigen presenting cells (APC) preparation

CFSE modification was done for 15 min at 37°C with 20x10^6^ cells/ml in a water bath. Cells were washed once by addition of a 4-fold volume of MACS buffer (0.5% fetal calf serum (FCS, Biochrome), 2 mM EDTA in PBS). TNBS modification (Sigma Aldrich, Cat# [92822](https://www.sigmaaldrich.com/DE/en/product/sial/92822?context=product)-1ML) was done afterwards with 20x10^6^ cells/ml and cells were washed three times in a 4-fold volume of MACS buffer.

## T cell antigen stimulation assay

Antibodies for T cell antigen stimulation assays and major histocompatibility complex *(*MHC) blocking experiments:

| **Antigen** | **Clone** | **Concentration** | **Company** | **Catalog number and/or identifier** |
| --- | --- | --- | --- | --- |
| CD40 | HB14 | 1 µg/ml | Miltenyi Biotec | Cat# 130-094-133; RRID:AB_10839704 |
| HLA-DR, -DP, -DQ | Tu39 | 10 µg/ml | BD Biosciences | Cat# 555556; RRID:AB_395938 |
| HLA-DR | Ac122 | 10 µg/ml | Miltenyi Biotec | Cat# 130-108-056; RRID:AB_2661330 |
| HLA-DR | L243 | 10 µg/ml | BioLegend | Cat# 307602; RRID:AB_314680 |
| HLA-ABC | W6/32 | 10 µg/ml | BioLegend | Cat# 311428; RRID:AB_2561492 |
| -(IgG2a isotype control) | MOPC-173 | 10 µg/ml | BioLegend | Cat# 400224; RRID:AB_2861018 |

## Antibody staining and flow cytometry analysis

Antibodies for flow cytometry were titrated to determine optimal dilutions and staining volumes.

| **Antigen** | **Clone** | **Fluorochrome conjugate** | **Company** | **Catalog number and/or identifier** |
| --- | --- | --- | --- | --- |
| CD3 | UCHT1 | AlexFluor® 700 | BioLegend | Cat# 300424; RRID:AB_493741 |
| CD4 | SK3 | BB700 | BD Biosciences | Cat# 566392; RRID:AB_2744421 |
| CD8 | SK1 | BV510 | BioLegend | Cat# 344732; RRID:AB_2564624 |
| CD45RA | HI100 | PE/Dazzle™ 594 | BioLegend | Cat# 304146; RRID:AB_2564079 |
| CCR7 | G043H7 | PE-Cy7 | BioLegend | Cat# 353226; RRID:AB_11126145 |
| CD154 | 5C8 | PE | Miltenyi Biotec | Cat# 130-113-607; RRID:AB_2751142 |
| CD137 | 4B4-1 | APC | BioLegend | Cat# 309810; RRID:AB_830672 |
| CD69 | FN50 | BV711 | BioLegend | Cat# 310944; RRID:AB_2566466 |
| CD14 | M5E2 | BV421 | BioLegend | Cat# 301830; RRID:AB_10959324 |
| CD19 | HIB19 | APC | BD Biosciences | Cat# 555415, RRID:AB_398597 |
| TNP | A19-3 | BV421 | BD Biosciences | Cat# 562601; RRID:AB_11153675 |
| dead cells | - | * | Thermo Fisher Scientific | Cat# L10119 |

*LIVE/DEAD™ Fixable Near-IR Dead Cell Stain Kit. Dead cell stain was added immediately before use to the antibody mix to stain cells simultaneously for surface markers and dead cells.

Stainings were performed in FACS buffer (PBS, 2 mM EDTA, 0.5% FCS). Our BD FACSAria III is equipped with three lasers (405 nm, 488 nm, 633 nm) and mirror/filter combinations for the simultaneous detection of 11 colors. For samples from 96- and 24-well plates, all events were acquired. For samples from 12- and 6-well plates, we collected 1 million total events and afterwards stored for remaining cells all events contained in the SSC single cell gate (see gating strategy, **Figure S4**).

## Single cell sort, expansion and restimulation of T cell clones

Additional functional antibody for single clone expansion and restimulation assays.

| **antigen** | **clone** | **concentration** | **company** | **catalog number and/or identifier** |
| --- | --- | --- | --- | --- |
| CD3 | OKT3 | 30 ng/ml (in feeder medium)  1 µg/ml (plate coating) | Miltenyi Biotec | Cat# 130-093-387; RRID:AB_1036144 |
| CD28 | 15E8 | 1 µg/ml | Miltenyi Biotec | Cat# 130-093-375; RRID:AB_1036134 |

Feeder cells were prepared by incubation of 50x10^6^ PBMC/ml with 50 µg/ml mitomycin C (Abcam) in PBS and 10 % FCS (Biochrome) for 30 minutes at 37°C with occasional mixing and extensive washing afterwards. Cells were kept in liquid nitrogen until use.

Positive specific clone restimulation was assumed if the following conditions were fulfilled: (a) the frequency of CD154+CD4+ or CD137+CD8+ cells was 3-times higher in antigen-stimulated samples compared to samples without antigen, (b) the frequency of CD154+CD4+ or CD137+CD8+ T cells after Phorbol-12-myristat-13-acetat (PMA)-Ionomycine stimulation was >30% in T cells without APC and (c) the count of CD154+CD4+ or CD137+CD8+ cells was >20 cells.

## Supplementary References

Zimmermann, J., Radbruch, A., and Chang, H.D. (2015). A Ca(2+) concentration of 1.5 mM, as present in IMDM but not in RPMI, is critical for maximal response of Th cells to PMA/ionomycin. *Eur J Immunol* 45**,** 1270-1273.
